# Genome-wide imputed differential expression enrichment analysis identifies trait-relevant tissues

**DOI:** 10.3389/fgene.2022.1008511

**Published:** 2023-01-06

**Authors:** Ammarah Ghaffar, Dale R. Nyholt

**Affiliations:** Statistical and Genomic Epidemiology Laboratory, School of Biomedical Sciences, Faculty of Health and Centre for Genomics and Personalised Health, Queensland University of Technology, Brisbane, QLD, Australia

**Keywords:** genome-wide association, complex traits, expression quantitative trait loci, bioinformatics, statistical genetics

## Abstract

The identification of pathogenically-relevant genes and tissues for complex traits can be a difficult task. We developed an approach named genome-wide imputed differential expression enrichment (GIDEE), to prioritise trait-relevant tissues by combining genome-wide association study (GWAS) summary statistic data with tissue-specific expression quantitative trait loci (eQTL) data from 49 GTEx tissues. Our GIDEE approach analyses robustly imputed gene expression and tests for enrichment of differentially expressed genes in each tissue. Two tests (mean squared z-score and empirical Brown’s method) utilise the full distribution of differential expression *p*-values across all genes, while two binomial tests assess the proportion of genes with tissue-wide significant differential expression. GIDEE was applied to nine training datasets with known trait-relevant tissues and ranked 49 GTEx tissues using the individual and combined enrichment tests. The best-performing enrichment test produced an average rank of 1.55 out of 49 for the known trait-relevant tissue across the nine training datasets—ranking the correct tissue first five times, second three times, and third once. Subsequent application of the GIDEE approach to 20 test datasets—whose pathogenic tissues or cell types are uncertain or unknown—provided important prioritisation of tissues relevant to the trait’s regulatory architecture. GIDEE prioritisation may thus help identify both pathogenic tissues and suitable proxy tissue/cell models (e.g., using enriched tissues/cells that are more easily accessible). The application of our GIDEE approach to GWAS datasets will facilitate follow-up *in silico* and *in vitro* research to determine the functional consequence(s) of their risk loci.

## 1 Introduction

Genome-wide association studies have been successfully applied to thousands of traits. However, single nucleotide polymorphisms (SNPs) identified *via* GWAS only explain a small fraction of heritability for most traits, and the genome-wide significant variants (*p* < 5 × 10^−8^) do not necessarily pinpoint the causal variants and genes (Manolio et al., 2009; Boyle et al., 2017). Moreover, the functional interpretation of GWAS variants remains largely unknown. Therefore, annotating the possible functional effect of GWAS variants is important to understanding their effect on a trait. Also, SNPs that are associated at a genome-wide *suggestive* threshold (i.e., 5 × 10^−8^ < *p* < 1 × 10^−5^) can nonetheless be truly associated with the trait and can be identified (implicated) by leveraging multi-omic data such as gene expression. Integration of GWAS studies with functional data, such as expression quantitative trait loci (eQTL), is one way to demonstrate that a GWAS variant within a particular region influences the expression of the gene (Stranger et al., 2007), and has the potential to implicate SNPs and genes *via* differential expression even at GWAS loci that do not reach genome-wide significance.

Complex traits have multiple genes involved in their aetiology and their pathogenic tissues or cell types are mostly uncertain or unknown. Identifying the likely pathogenic trait-relevant tissue(s) is critical for developing systems to explore gene regulatory mechanisms that contribute to the trait. In recent years, a lot of data and research has been published that provides insight into which parts of the genome are active in a range of tissues and cell types—for example, which parts of the genome are accessible (e.g., region of open chromatin) and which genes are expressed (Feingold and Pachter, 2004; Kundaje et al., 2015; Ward et al., 2015). Combining this type of information with GWAS data offers the potential to identify pathogenic tissues and cell types for complex traits.

The majority of GWAS risk variants are non-coding and are thus expected to impact the expression of the gene by altering its regulation (Ward and Kellis, 2012). eQTL analysis is the most common approach to evaluating the effect of variants present in the human genome on gene expression (Morley et al., 2004; Grundberg et al., 2012; Westra et al., 2013). However, eQTL studies are expensive and often limited by the availability of relevant tissue. This limitation has been addressed by the Genotype-Tissue Expression (GTEx) project that hosts gene expression, eQTL and genotype data from the same individuals across different tissues (Ward et al., 2015). The eQTL status of a trait-associated SNP provides a potential link between GWAS loci and genes mediating potential genetic effects (Nicolae et al., 2010). Recently, several methods such as MetaXcan (Barbeira et al., 2016) have been developed which integrate eQTL information with GWAS to impute genetically regulated trait-associated gene expression. These methods also have the advantage of combining small effects of multiple cis-SNPs at the gene level, thus reducing the multiple test burden compared to testing all individual SNPs across the genome.

Linkage disequilibrium (LD) score regression applied to specifically expressed genes (LDSC-SEG) is another approach that attempts to identify trait-relevant tissue and cell types using GWAS summary statistics and gene expression data (Finucane et al., 2018). In this approach, the authors calculated a t-statistic for each gene expressed in a specific tissue versus all other tissues and identified the top 10% of genes ranked by the t-statistic. A 100 kb window was added around the top 10% of genes and LDSC score regression was performed to estimate SNP-based heritability for each tissue-gene set. Using LDSC-SEG, Finucane et al. (2018) were able to find tissue (heritability) enrichments for several GWAS traits using gene expression data from five different sources including GTEx.

In this study, a novel approach named genome-wide imputed differential expression enrichment (GIDEE) was developed, to prioritise tissues relevant to the trait’s regulatory architecture by combining GWAS summary statistic data with tissue-specific eQTL data. This method can be viewed as an extension of transcriptome-wide association studies (TWAS). GIDEE utilises the top 50th percentile of accurately imputed gene expression in downstream enrichment analyses. For each tissue, the enrichment of trait-associated differential expression is evaluated using four tests. Two tests utilise the distribution of differential expression *p*-values across all genes, and two tests assess the proportion of genes with tissue-wide significant differential expression. The GIDEE approach was able to prioritise trait-relevant tissues for the training dataset in the top 3 of the 49 GTEx tissues. For the test datasets, GIDEE provided important prioritisation of tissues with regulatory mechanisms (eQTLs) associated with the trait. These tissues could be the pathogenic tissues or accessible proxy tissues that will aid in the design of follow-up functional laboratory studies aimed at characterising GWAS risk loci.

## 2 Materials and methods

**FIGURE 1:**
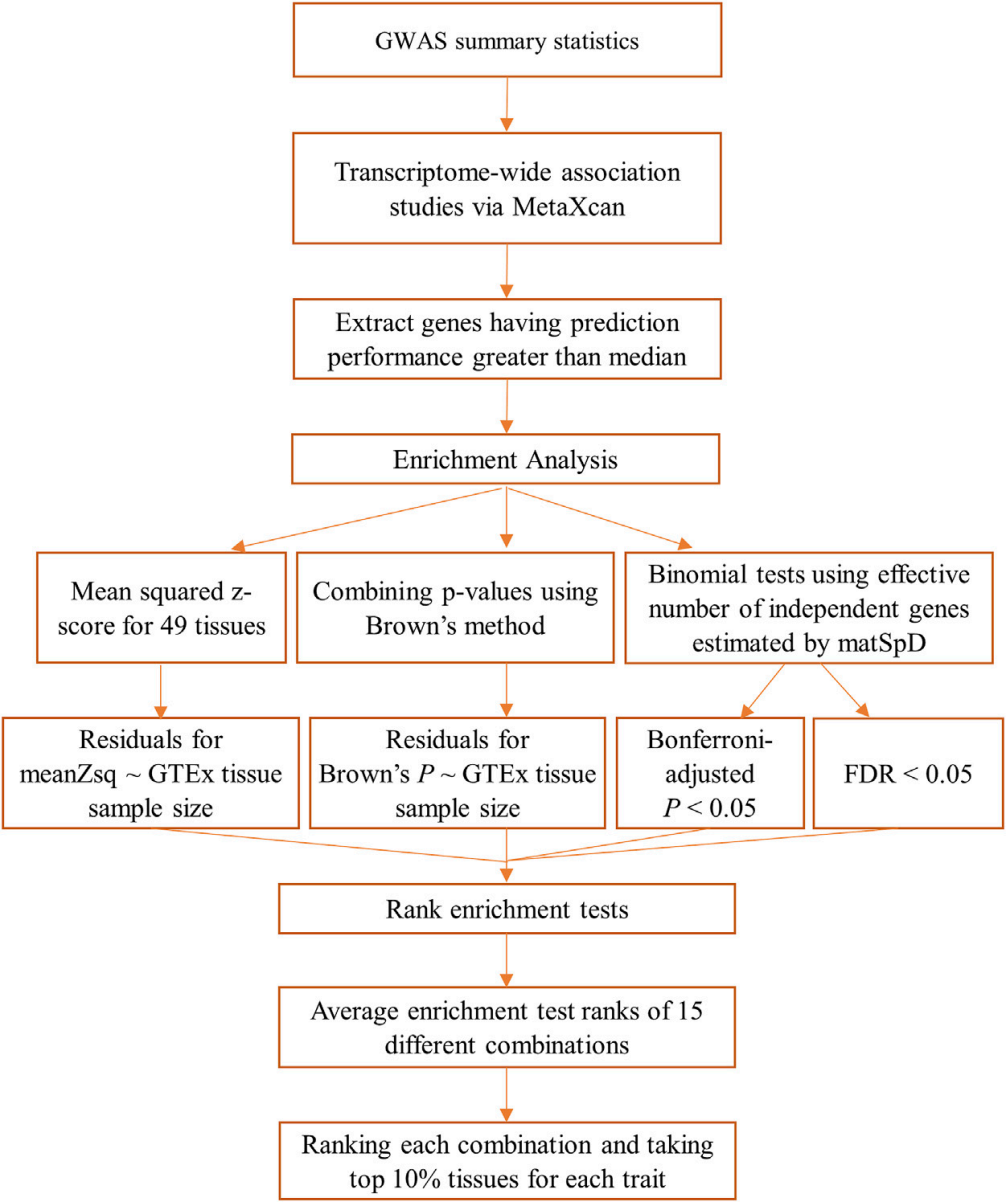
Overview of the methodology followed for each of the 29 GWAS datasets used in this study.

An overview of the methods followed for the GIDEE approach of the 29 GWAS traits used in this study is provided in Figure 1. The first step was to access GWAS summary statistics for 29 traits from multiple resources. Once GWAS summary statistics were pre-processed and harmonised, TWAS was performed for all traits and all 49 GTEx tissues. This was followed by enrichment analysis including only the genes having prediction performance better than the median prediction performance. Four enrichment tests were performed and 49 GTEx tissues were ranked according to 15 different combinations of these primarily four enrichment tests. Tissues were prioritised for each trait based on differential gene enrichment tests. Each step is described in detail in the following sections.

### 2.1 Datasets

#### 2.1.1 GWAS summary statistics datasets

A total of 29 GWAS datasets were analysed (Table 1; Table 2). Nine of these GWAS datasets were used as training datasets. They are called training datasets because biological evidence to support the involvement of a specific tissue in its pathogenesis exists. For example, T-cells lymphocytes play an important role in the pathogenesis of asthma (Lloyd and Hessel, 2010) and eczema (Tamaki and Nakamura, 2001). Similarly, the spleen, an organ that plays an important role in the body’s immune response, is known to be associated with several gastrointestinal diseases. Functional hyposplenism, loss of function of the spleen, is associated with ulcerative colitis, Crohn’s disease and inflammatory bowel disease (Ryan et al., 1978). Breast and prostate are associated with the pathogenesis of breast and prostate cancer, respectively. Similarly, the pancreas is associated with type 2 diabetes (Galicia-Garcia et al., 2020). In type 2 diabetes, the body builds up insulin resistance and more insulin is needed to bring down blood glucose levels. As a result, the pancreas needs to produce more insulin than it would normally need to. Similarly, adipose tissue distribution is associated with the waist-to-hip ratio (Daniel et al., 2003). Table 1 shows the trait in the training dataset along with the respective tissue that is involved in the pathogenicity of the trait (with references). Therefore, these datasets were termed “training” datasets as they were used to test and calibrate the GIDEE approach, in particular, the differential expression enrichment tests. The resulting approach was then applied to 20 “test” datasets, whose pathogenic tissues or cell types are uncertain or unknown, to prioritise their likely trait-relevant pathogenic tissues and tissues related to the regulatory mechanism of the trait. However, it is important to note that although we do not know the exact biological tissue(s) involved in the test datasets, a general biological system has been implicated. For example, we know attention deficit hyperactivity disorder (ADHD), Alzheimer’s disease, autism spectrum disorder, bipolar disorder, depressive symptoms, neuroticism, and schizophrenia are neurological disorders (implicating the nervous system). Similarly, blood pressure GWASs (e.g., diastolic blood pressure, hypertension, systolic blood pressure) can be grouped and related to vascular function (implicating the circulatory/cardiovascular system).

**TABLE 1:** Detail of all GWASs used as the training dataset.

Trait name	Asthma	Breast cancer	Eczema	Prostate cancer	Ulcerative colitis	Waist–hip ratio (BMI adjusted)	Crohn’s disease	IBD	Type 2 diabetes
GWAS sample size	361,141	247,173	361,141	140254	27,432	458,417	20,883	34,652	361,141
Cases	41,934	133,384	9321	79148	6968		5,956	12,882	2292
Controls	319,207	113,789	351820	61106	20464		14,927	21,770	358849
Consortium	United Kingdom Biobank	BCAC	United Kingdom Biobank	PRACTICAL	N/A	United Kingdom Biobank	N/A	N/A	United Kingdom Biobank
GWAS type	Binary	Binary	Binary	Binary	Binary	Continuous	Binary	Binary	Binary
Tissue	Cells EBV transformed lymphocytes	Breast	Cells EBV transformed lymphocytes	Prostate	Spleen	Adipose subcutaneous	Spleen	Spleen	Pancreas
References	Lloyd and Hessel, (2010)	Boyd et al. (2010)	Tamaki and Nakamura, (2001)	Mohler et al. (2010)	Muller et al. (1993)	Daniel et al. (2003)	Corazza and Gasbarrini, (1983)	Ryan et al. (1978)	Ozougwu et al. (2013)
Adipose Subcutaneous	4316	4307	4316	4315	4307	4316	4305	4307	4316
Adipose Visceral Omentum	3661	3656	3661	3661	3651	3661	3650	3651	3661
Adrenal Gland	2415	2410	2415	2415	2410	2415	2409	2410	2415
Artery Aorta	3793	3786	3793	3793	3785	3793	3783	3785	3793
Artery Coronary	2016	2013	2016	2015	2013	2016	2013	2013	2016
Artery Tibial	4299	4293	4299	4298	4293	4299	4292	4293	4299
Brain Amygdala	1388	1386	1388	1388	1384	1388	1383	1384	1388
Brain Anterior cingulate cortex BA24	1767	1766	1767	1767	1764	1767	1763	1764	1767
Brain Caudate basal ganglia	2495	2491	2495	2494	2487	2495	2487	2487	2495
Brain Cerebellar Hemisphere	2870	2866	2870	2870	2865	2870	2864	2865	2870
Brain Cerebellum	3389	3383	3389	3390	3384	3389	3382	3383	3389
Brain Cortex	2740	2736	2740	2741	2736	2740	2734	2736	2740
Brain Frontal Cortex BA9	2272	2269	2272	2272	2266	2272	2266	2266	2272
Brain Hippocampus	1839	1836	1839	1839	1836	1839	1836	1836	1839
Brain Hypothalamus	1821	1819	1821	1821	1816	1821	1816	1816	1821
Brain Nucleus accumbens basal ganglia	2419	2416	2419	2419	2414	2419	2413	2414	2419
Brain Putamen basal ganglia	2213	2209	2213	2213	2208	2213	2207	2208	2213
Brain Spinal cord cervical c-1	1622	1620	1622	1621	1618	1622	1618	1618	1622
Brain Substantia nigra	1277	1275	1277	1275	1273	1277	1273	1273	1277
Breast Mammary Tissue	3223	3218	3223	3223	3214	3223	3213	3214	3223
Cells Cultured fibroblasts	4458	4454	4458	4458	4450	4458	4449	4450	4458
Cells EBV-transformed lymphocytes	1448	1447	1448	1448	1444	1448	1444	1444	1448
Colon Sigmoid	3078	3074	3078	3078	3068	3078	3068	3068	3078
Colon Transverse	3145	3139	3145	3145	3139	3145	3138	3139	3145
Esophagus Gastroesophageal Junction	3138	3134	3138	3138	3134	3138	3132	3133	3138
Esophagus Mucosa	4251	4246	4251	4251	4243	4251	4242	4243	4251
Esophagus Muscularis	4107	4104	4107	4106	4095	4107	4094	4095	4107
Heart Atrial Appendage	3314	3310	3314	3314	3306	3314	3305	3306	3314
Heart Left Ventricle	3002	2999	3002	3002	2997	3002	2996	2997	3002
Kidney Cortex	818	817	818	818	815	818	815	815	818
Liver	1881	1879	1881	1881	1877	1881	1877	1877	1881
Lung	3975	3970	3975	3976	3968	3975	3966	3968	3975
Minor Salivary Gland	1455	1453	1455	1454	1451	1455	1451	1451	1455
Muscle Skeletal	3786	3783	3786	3786	3782	3786	3781	3782	3786
Nerve Tibial	4997	4989	4997	4997	4987	4997	4985	4987	4997
Ovary	1788	1785	1788	1788	1785	1788	1784	1785	1788
Pancreas	2943	2936	2943	2942	2937	2943	2936	2937	2943
Pituitary	2836	2832	2836	2836	2830	2836	2829	2830	2836
Prostate	2145	2141	2145	2146	2140	2145	2140	2140	2145
Skin Not Sun Exposed Suprapubic	4318	4309	4318	4316	4311	4318	4309	4311	4318
Skin Sun Exposed Lower leg	4641	4634	4641	4641	4631	4641	4630	4631	4641
Small Intestine Terminal Ileum	1829	1825	1829	1829	1827	1829	1827	1827	1829
Spleen	2881	2873	2881	2880	2876	2881	2874	2876	2881
Stomach	2569	2566	2569	2568	2563	2569	2562	2562	2569
Testis	4976	4967	4976	4976	4965	4976	4964	4965	4976
Thyroid	4817	4814	4817	4817	4812	4817	4810	4812	4817
Uterus	1266	1265	1266	1266	1262	1266	1262	1262	1266
Vagina	1276	1272	1276	1276	1273	1276	1273	1273	1276
Whole Blood	3620	3616	3620	3620	3614	3620	3613	3614	3620

**TABLE 2:** Detail of all GWASs used as the test dataset.

Trait name	N	Cases	Controls	Consortium	Type
ADHD	55,374	20,183	35,191	Brainstorm, PGC	Binary
Alzheimer’s disease	54,162	17,008	37,154	Brainstorm, IGAP	Binary
Autism Spectrum Disorder	46,351	18,382	27,969	Brainstorm, PGC	Binary
Bipolar Disorder	51,710	20,352	31,358	Brainstorm, PGC	Binary
Depressive symptoms	161,460			SSGAC	Continuous
Diastolic blood pressure	340,162			United Kingdom Biobank	Continuous
Ischemic stroke and subtypes	74,339	12,389	62,004	Brainstorm, ISGC	Binary
Fasting Glucose	46,186			MAGIC	Continuous
HDL	99,900			N/A	Continuous
Heel T-Score	445,921			United Kingdom Biobank	Continuous
Height	360,388			United Kingdom Biobank	Continuous
Hypertension	361,141	93,560	267,581	United Kingdom Biobank	Binary
LDL	95,454			N/A	Binary
Migraine (all subtypes)	375,752	59,674	316,078	Brainstorm, IHGC	Binary
Neuroticism	170,911			SSGAC	Continuous
Schizophrenia	306,011	69,369	236,642	Brainstorm, PGC	Binary
Smoking Status	457,683			United Kingdom Biobank	Continuous
Systolic blood pressure	340,159			United Kingdom Biobank	Continuous
Triglycerides	96,598			N/A	Continuous
Years of Education	394,792			SSGAC	Continuous

Details of the training and test datasets are provided in Table 1 and Table 2, respectively. All GWAS datasets were pre-processed to harmonise the SNP summary statistics with respect to their effect allele, non-effect allele, and chromosome position (i.e., their base pair (bp) position was “lifted over” to genome build 38) to ensure compatibility with the genetic (gene expression) predictor models from GTEx version 8. The datasets contained a mix of binary and continuous traits.

#### 2.1.2 Gene expression dataset

The gene expression and eQTL datasets were obtained from GTEx. The GTEx project aimed to establish a comprehensive database and resource that enables the study of tissue-specific gene expression. The pilot study for the GTEx utilised 1,641 samples of 43 tissues from 175 donor individuals to perform RNA sequencing, gene expression analysis across tissues (53,934 genes in total), eQTL analysis (single tissue and multiple tissue eQTL analysis), allele-specific expression analysis, and splicing QTLs analysis (Ward et al., 2015). The current release of GTEx version 8 (v8) has data for 54 tissues obtained from 948 donors summing to a total number of 17,382 samples. Genotype and eQTL data were available for 49 tissues (N ≥ 70 samples) from 838 donors summing to a total number of 15,201 samples. Fully processed, filtered, and normalised gene expression matrices (in BED format) for each tissue were downloaded from GTEx v8 portal (https://gtexportal.org/home/).

### 2.2 Gene-trait association (MetaXcan)

MetaXcan was used to compute gene-trait association (differential gene expression) in 49 human tissues from GTEx v8. MetaXcan uses a set of reference individuals whose gene expression and genotyping have been measured for the same individuals. The authors of MetaXcan utilised the GTEx data, adjusted for sex and experimental/population confounders, and used an elastic net model to calculate expression weights for each SNP present ± 1 Mb of the gene (Barbeira et al., 2016). These weights for each tissue are available in the form of SQLite weight files available on predictdb.org (Gamazon et al., 2015; Barbeira et al., 2018; Barbeira et al., 2021). The GTEx v8 elastic net prediction models “elastic_net_eqtl.tar” containing weights of the predictor SNPs on each gene within each tissue along with a single tissue covariance file were retrieved from predictdb.org on 11/03/2020.

To derive a quality metric for each gene model, the authors used 10-fold cross-validation to compare imputed gene expression results with the original gene expression data available *via* GTEx. This metric is labelled as “pred.perf.R2” which is the square of the correlation measure between the imputed and original gene expression. Thus, the higher the value of “pred.perf.R2” the higher the accuracy of gene-trait association. Therefore, to ensure our enrichment tests use robust estimates of genetically predicted differential expression, for each tissue, enrichment analysis was restricted to genes having “pred.perf.R2” greater than the median of “pred.perf.R2” (i.e., the top 50th percentile of accurately imputed genes from each tissue for each trait were used).

The genetic prediction weights were used to impute gene expression (which is unobserved in a typical GWAS) by estimating the genetically determined component using elastic net prediction models. The 1000 genomes project data was used as the LD reference. The imputed gene expression was then tested for association with the GWAS trait. The association is quantified *via* a z-score. Briefly, the z-score represents differential expression, where a positive z-score indicates an increased expression of a gene is associated with the trait (i.e., increased risk for the GWAS trait). A negative z-score means a reduced expression of a gene is associated with the trait. The statistical significance of the association is expressed as a z-score and its corresponding two-sided *p*-value.

### 2.3 Enrichment analysis

Four tests were used to assess differential expression enrichment in each tissue. Two tests utilised the distribution of differential expression *p*-values across all genes, while two tests assessed the proportion of genes with tissue-wide significant differential expression.

#### 2.3.1 Differential expression z-score adjusted for GTEx sample size

The average significance of differential expression, quantified as the mean squared z-score across all genes, was the first measure of enrichment that utilised the distribution of differential expression *p*-values across all genes. The square was taken to remove positive and negative signs. Here, the need to account for differential power to detect associations for the different GTEx tissue sample sizes was recognised. Supplementary Table S1 shows the GTEx v8 tissues, their sample size, and the system category to which they belong. Hence, a linear regression model (lm function in R) was used and the mean z-squared value for each tissue was regressed on the GTEx tissue sample size. The distance along the y-axis from the fitted line to the observed point for each tissue (residual) was noted. The larger the distance from the fitted line (i.e., the larger the residual), the more the tissue was enriched for differentially expressed genes.

#### 2.3.2 Combining dependent p-values (Brown’s method) adjusted for GTEx sample size

The second measure of enrichment combined all imputed differential expression *p*-values using the empirical Brown’s method. Brown’s method was chosen because it takes into account the dependency of *p*-values, whereas other methods such as Fisher’s method and Stouffer’s method assume *p*-values to be independent and uncorrelated (Poole et al., 2016). Brown’s method uses a correlation factor ‘c’ which is the ratio of the degree of freedom used by Fisher’s method (considering all genes as independent) and the re-scaled degrees of freedom used by Brown’s method (taking into account the correlation within genes in each tissue). Brown’s method uses an empirical cumulative distribution function derived directly from data. Brown’s method combines equally weighted dependent *p*-values assuming normally distributed underlying data. The package implementing Brown’s method is available in R which requires a data matrix (from which dependency is estimated) and *p*-values as input. Therefore, gene expression matrices for all 49 tissues from GTEx v8 were downloaded. For each tissue, the expression values for genes, whose differential expression was imputed by MetaXcan, were extracted from the GTEx gene expression matrices and used, along with the differential expression *p*-values from MetaXcan, as input to Brown’s method. Analogous to the mean z-squared enrichment test, Brown’s *p*-values were adjusted for GTEx tissue sample size using a linear regression model (lm function in R). The distance along the y-axis from the fitted line to the observed point for each tissue (residual) was noted. The larger the distance (residual) the more the tissue was enriched for differentially expressed genes.

#### 2.3.3 Binomial tests for the effective number of independent genes with two thresholds (Bonferroni and FDR)

The last two enrichment measures used one-sided binomial tests to see if the proportion of differentially expressed genes in each tissue was greater than expected (*p* < 0.05). Two thresholds of tissue-wide significant differential expression were used. First, for each tissue, the effective number of independent genes was estimated to account for the substantial covariance in expression across genes—i.e., multiple-test adjustment using the total raw number of genes would be too stringent and would not reflect the true biology. The effective number of independent genes analysed for differential expression in each tissue was estimated using matrix spectral decomposition (matSpD) (Nyholt, 2004). The matSpD approach estimates the effective number of independent variables (in this case genes) by analysing the eigenvalues produced from the spectral decomposition of a correlation matrix. The expression values for genes whose differential expression was predicted by MetaXcan were extracted from normalised gene expression matrices obtained from GTEx. Briefly, a gene expression pairwise Pearson correlation matrix was generated using R and used as input to the matSpD.R script (downloaded from https://drive.google.com/open?id=1-r-HWsKOD8NfbOG4C4SFIwjj8yYze2Zu). The output is an effective number of independent genes along with a *p*-value to effectively control for type 1 error at 5%. The estimated effective number of independent genes was used as “*n: number of trials*” in the binom.test function in R and to calculate a tissue-wide significant threshold adjusted for multiple testing (i.e., *p* = 0.05/effective number of independent genes). Genes having a differential expression *p*-value less than the matSpD-adjusted significance threshold were considered to have tissue-wide significant differential expression. The effective number of independent genes with tissue-wide significant differential expression was estimated *via* matSpD and was used as the “*x: number of successes*” in the binom.test function in R. The observed proportion of enriched genes was thus calculated as *x: number of successes* divided by *n: number of trials*. The null (expected) proportion was calculated as the sum of all independent genes having differential expression less than the matSpD-adjusted *p*-value and subtracting the number of independent genes less than the matSpD *p*-value present in tissue i divided by the sum of the number of independent genes across all tissues and subtracting the number of independent genes present in tissue i . This approach assessed whether tissue *i* had a significantly increased proportion of tissue-wide significant (*p* < 0.05) differentially expressed genes compared to the mean of the other 48 tissues. We repeated the same procedure for all tissues *i* ranging from 1 to 49.

$$null\left[i\right]=\frac{sum\left(success\right)-success\left[i\right]}{sum\left(trial\right)-trial\left[i\right]}$$ (1)

The fourth and final enrichment test used a binomial test with a less stringent tissue-wide significant differential expression threshold, where the *p*-values were adjusted for multiple testing using the Benjamini & Hochberg False Discovery Rate (FDR) procedure implemented in the *p.adjust* function from the *stats* base R package, with option *method= “BH”*. Genes having a differential expression *p.adjust p*-value (FDR) less than 0.05 were considered to have tissue-wide significant differential expression. The effective number of independent genes with tissue-wide significant differential expression (FDR < 0.05) was subsequently estimated *via* matSpD and used as the *x: number of successes* in the binom.test function in R. The null proportion was calculated as in (1) analogous to the first binomial test. The second binomial test assessed whether tissue *i* had a significantly increased proportion of tissue-wide significant (FDR < 0.05) differentially expressed genes compared to the mean of the other 48 tissues.

Both binomial tests were not additionally adjusted for GTEx tissue sample size because the *x: number of successes* and *n: number of trials* estimates are calculated specific to each tissue and are thus related to the GTEx tissue sample size.

### 2.4 Rank and average of the rank of enrichment methods

Given the utilised enrichment measures use and examine different sections of the differential expression *p*-value distribution, for each GWAS dataset we examined the rank of each tissue according to the enrichment *p*-values from the four enrichment methods.

#### 2.4.1 Ranking of enrichment methods

Mean squared z-score and Brown’s method *p*-value residuals were ranked in ascending order (i.e., the larger the residual the higher the tissue’s rank). The binomial test *p*-values were ranked in descending order (i.e., the smaller the *p*-value the higher the tissue’s rank).

#### 2.4.2 Average of different combinations of enrichment methods followed by ranking of all combinations

In addition to assessing tissue rankings for the four enrichment measures in the training datasets, we assessed all possible combinations of the four rankings by estimating the average ranks of the combined ranks. Supplementary Table S2 shows all 15 possible combinations which were used for ranking. The higher the average rank of tissue, the higher the evidence for differential expression enrichment and the more likely the tissue is pathogenically relevant to the GWAS datasets.

## 3 Results

### 3.1 Analysis of training datasets across 49 GTEx tissues

For nine training datasets, the tissue that plays a major role in the pathogenicity of the trait is known.

Table 1 shows the number of genes in the top 50th percentile of accurately imputed genes from each tissue for each trait that were used in downstream enrichment analyses. Supplementary Table S3 shows R^2^, adjusted R^2^, and *p*-value describing the variance in mean squared z-score and Brown’s *p*-value explained by GTEx tissue sample size. Supplementary Table S3 shows that for eight of the nine training datasets, the variance in mean squared z-score values was significantly (*p* < 0.05) related to GTEx tissue sample size. Similarly, for six of the nine training datasets, the variance in Brown’s *p*-values was related to GTEx tissue sample size. For both tests, the number of genes found to be differentially expressed was proportional to the tissue sample size. It is important to note that GTEx tissues with larger eQTL sample sizes impute differential expression for more genes because they have more power to model a relationship between genotypes and expression. Hence, there is greater power to detect differentially expressed genes in tissues with larger sample sizes. It is also important to note that Brown’s method takes into account the correlation in expression that exists between genes in each tissue. Therefore, when combining *p*-values using Brown’s method, the tissue sample size is partly taken into consideration; however, residual analysis using the lm function in R still indicated a correlation between differential gene expression and GTEx sample size. The importance of such sample-size adjustment was even more evident for the mean squared z-score test—which was expected, given the mean estimate does not take into account correlation among genes within each tissue, so the relationship with sample size would be more pronounced compared to the Brown’s test.

**FIGURE 2:**
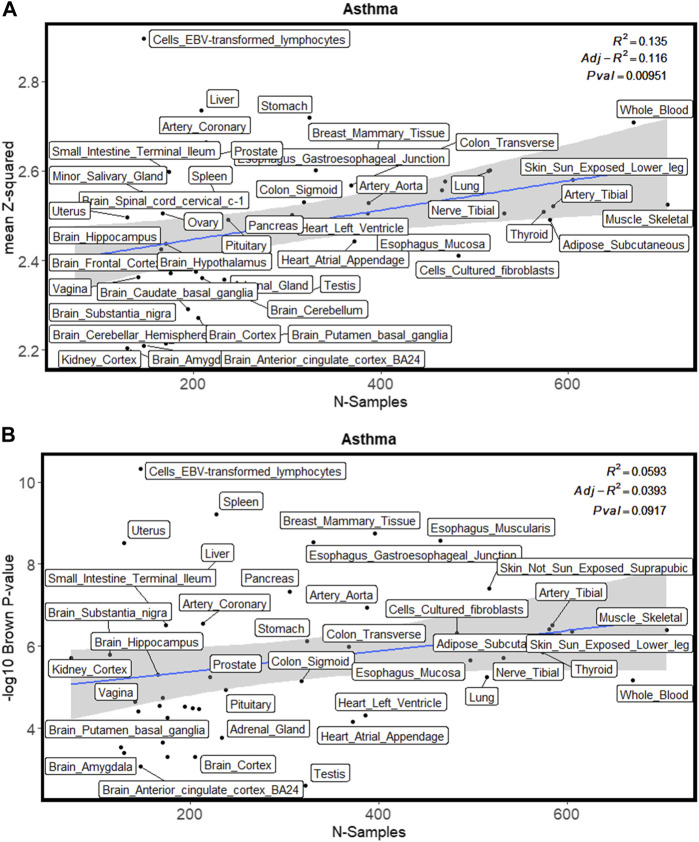
Enrichment methods used in this study for one of the training GWAS (asthma) datasets. **(A)** shows the enrichment with a mean squared z-score. **(B)** shows enrichment with Brown’s *p*-value.

**FIGURE 3:**
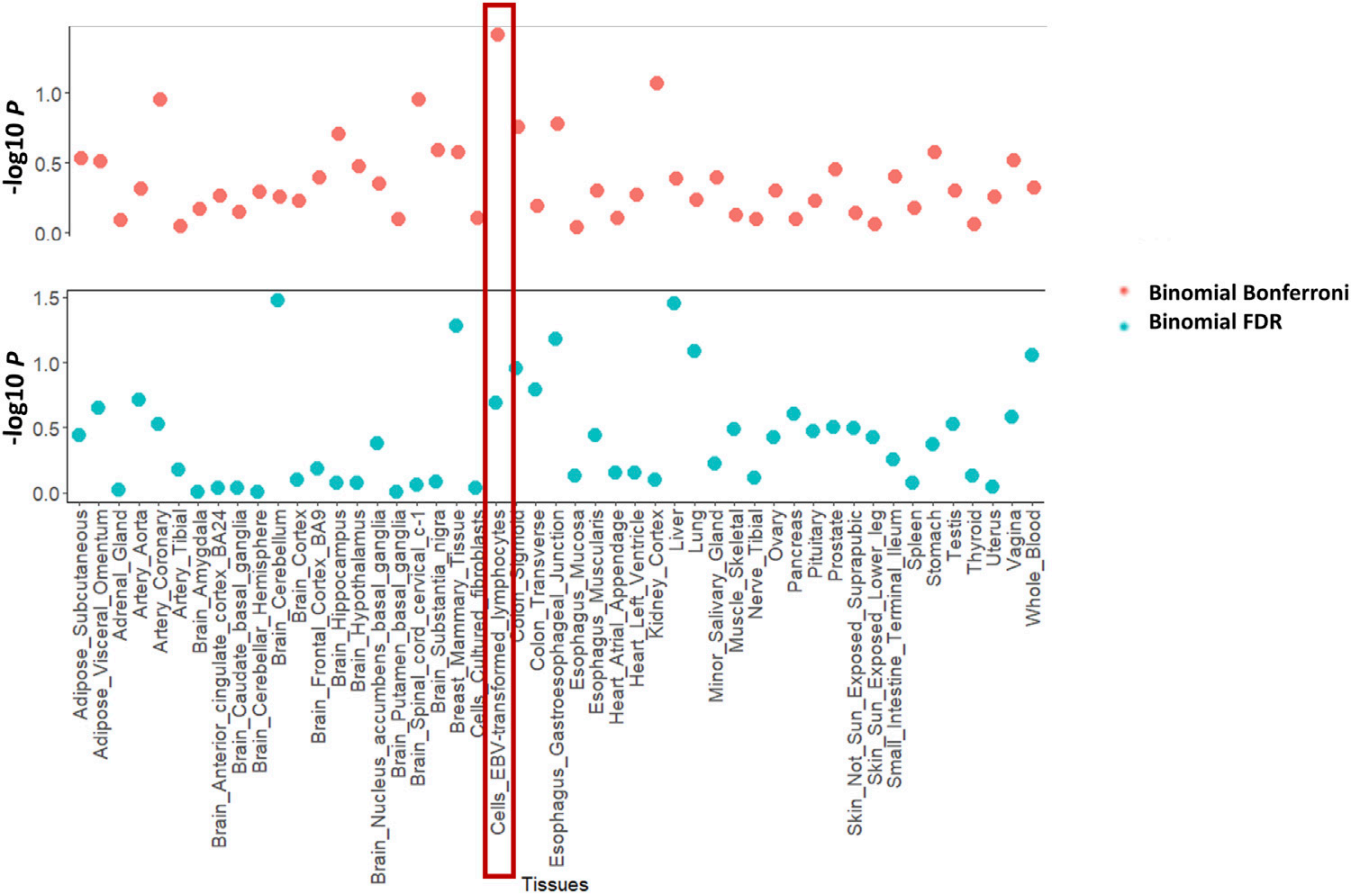
Enrichment methods used in this study for one of the training GWAS datasets (asthma). It shows the enrichment with the binomial test with Bonferroni and FDR threshold.

Figure 2 shows the enrichment test results for the asthma GWAS training dataset. Figure 2A shows the linear regression plot of the mean squared z-score against the GTEx sample size for all tissues. The blue line shows the best fit through the data. Cells_EBV_transformed_lymphocytes is the furthest tissue from the fitted line, thus implying that it had the highest enrichment of differentially expressed genes (as represented by z-score). Whole blood is one of the tissues having a large sample size because it is easily accessible. This results in more genes whose differential expression is imputed within this tissue. If not adjusted with tissue sample size, whole blood would be in the top 5 ranked tissues for asthma. This nicely exemplifies the importance of adjusting for GTEx tissue sample size. Similarly, Figure 2B shows that cells_EBV_transformed_lymphocytes had the highest enrichment of genes differentially expressed (as represented by Brown’s *p*-value). Figure 3 shows the proportion of differentially expressed genes present in cells_EBV_transformed_lymphocytes is highest as compared to other 48 tissues with strict threshold Bonferroni while using FDR threshold cells_EBV_transformed_lymphocytes was ranked 10th (Supplementary Table S4). Supplementary Table S5 contains raw values from each enrichment test for traits in the training datasets including Brown’s *p*-value, the effective number of independent genes calculated by matSpD, and the number of genes that were tested in the binomial tests. Supplementary Table S6 contains residuals for the Brown’s *p*-value and mean squared z-score after adjusting for GTEx sample size. Tissues were ranked based on these residuals as explained in the methods section. The file “Supplementary Figure S1” shows the plots for the mean squared z-score for each training dataset trait. The file “Supplementary Figure S2” shows plots for Brown’s *p*-values adjusted with GTEx sample size.

The file “Supplementary Figure S3” shows plots containing binomial test *p*-values using genes crossing the Bonferroni threshold (binomial_MatSpD) and FDR (binomial_FDR) threshold for tissue-wide significant differential expression (also see Supplementary Table S5 for raw values). For the four enrichment tests and their combinations (Supplementary Table S2), Supplementary Table S4 shows the individual and average rank of the known pathogenic tissue for the GWAS training datasets (specified in Table 1). Supplementary Table S7 contains the ranks for all tissues for all GWAS training datasets for the enrichment tests. Table 3 shows the range of unadjusted mean squared z-score, Brown’s *p*-value, and the tissues that crossed binomial Bonferroni and FDR *p* < 0.05. It is important to note that for six traits (asthma, breast cancer, eczema, prostate cancer, waist-hip ratio and IBD) all the tissues were significantly enriched for differentially expressed genes.

**TABLE 3:** Unadjusted mean squared z-score, Brown’s *p*-value and tissues crossing nominal Bonferroni and FDR *p* < 0.05 for the training dataset.

Traits	z-score across all 49 tissues	Brown’s *p*-value across all 49 tissues	Binomial Bonferroni *p*-value < 0.05	Binomial FDR *p*-value < 0.05
Asthma	2.18—2.89	4.77 × 10^−11^—0.0024	Cells EBV transformed lymphocytes	Brain Cerebellum
				Liver
Breast Cancer	1.94—2.59	1.96 × 10^−37^—2.99 × 10^−10^	Minor Salivary Gland	Liver
			Vagina	Heart Left Ventricle
				Minor Salivary Gland
Eczema	2.17—3.15	8.64 × 10^−13^—0.00094		Whole Blood
Prostate Cancer	1.59—2.388	1.43 × 10^−06^—0.022	Brain Amygdala	
Ulcerative Colitis	1.49—1.94	0.00020—0.109		Whole Blood
				Spleen
Waist–hip ratio (BMI adjusted)	3.17—4.66	1.02 × 10^−26^—1.84 × 10^−06^	Vagina	Adipose Subcutaneous
			Ovary	Breast Mammary Tissue
			Uterus	Cells Cultured fibroblasts
				Liver
				Muscle Skeletal
Crohn’s Disease	1.27—1.75	0.0015—0.1269	Uterus	Whole Blood
IBD	1.60—2.06	1.66 × 10^−05^—0.048	Uterus	Whole Blood
				Cells EBV transformed lymphocytes
Type 2 Diabetes	1.33—1.88	0.00032—0.058	Heart Left Ventricle	Heart Left Ventricle
			Minor Salivary Gland	Heart Atrial Appendage
				Minor Salivary Gland

Supplementary Table S4 shows that the SZBP combination (i.e., an average of the rank of the mean squared z-score and Brown’s *p*-value) is the overall smallest (highest-ranked) enrichment measure, implying that the combination of these two enrichment measures performed best in implicating the known pathogenic tissue (as described in Table 1) for the GWAS training dataset traits. Moreover, it is worth noting that the top-performing tests, according to average rank, were combinations containing Brown’s method. It is also to be noted that for the training dataset, the trait-relevant tissue was always in the top 3. For asthma, eczema, Crohn’s disease, inflammatory bowel disease and type 2 diabetes, the respective (expected) pathogenic tissue was ranked first. For prostate cancer, ulcerative colitis, and waist-hip ratio, the respective pathogenic tissue was ranked second. While for breast cancer, the respective pathogenic tissue was ranked third.

### 3.2 Analysis of test datasets

Once it was established that the SZBP combination was the best-performing method in the training datasets, the GIDEE approach (utilising the SZBP combination) was applied to the 20 test datasets. Supplementary Table S8 shows the range of unadjusted mean squared z-score and Brown’s *p*-value for the test traits. Although some support for biologically-related systems exists for the test datasets, these traits lack robust and validated biological evidence implicating a specific tissue in its pathogenesis. It was therefore reassuring to observe that the GIDEE approach ranked tissues higher (among the top 10% of GTEx tissues) from biologically-relevant systems compared to tissues from other systems. Also, although the training datasets had their trait-relevant tissues ranked in the top 3, for the test datasets, we highlight the top 5 tissues (top 10%) of the 49 tissues. Highlighting the top 5 tissues aligns well with gene expression profiling in GTEx, which showed that approximately a third of eQTL effects were estimated to be active in all or almost all tissues, while a fifth of eQTL effects were active in five or fewer tissues (Flynn et al., 2022). Therefore, of the genes imputed from GTEx eQTL data, approximately a third may be imputed in all tissues—and thus provide minimal insight into tissue ranking/prioritisation, while a fifth will be imputed in five or fewer tissues. Further support for highlighting the top 5 enriched tissues was provided by the results from the test datasets. For example, for neurological traits, brain tissues from GTEx were ranked among the top 10% of tissues. For hypertension, blood pressure traits, and migraine, artery tissues were ranked among the top 10% of tissues. Table 4 shows the top 10% of tissues prioritised as being enriched for candidate causal regulatory effects for the test traits. Supplementary Table S9 contains ranks for each tissue for all test datasets. For the test datasets, we found enrichment in tissues that recapitulate known biology of traits even if the pathogenic tissue(s) are unknown or unclear (i.e., tissues from biologically relevant systems were highly ranked).

**TABLE 4:** The top 10% of tissues prioritised as having candidate causal regulatory effects for the test datasets.

Trait	Combination 5: SZBP	System
ADHD	liver, uterus, breast mammary tissue, prostate, pituitary, brain substantia nigra	Digestive, CNS, Endocrine
Alzheimer Disease	artery aorta, liver, esophagus gastroesophageal junction, skin not sun exposed suprapubic, brain nucleus accumbens basal ganglia	Cardiovascular, Digestive, CNS
Autism Spectrum Disorder	artery coronary, brain cerebellum, breast mammary tissue, artery aorta, brain cerebellar hemisphere	Cardiovascular, CNS
Bipolar	pancreas, esophagus gastroesophageal junction, spleen, artery aorta, stomach	Digestive, Cardiovascular, Blood/Immune
Depressive Symptoms	liver, artery coronary, esophagus gastroesophageal junction, pituitary, ovary, brain hippocampus	Digestive, Cardiovascular, CNS, Endocrine
Diastolic Blood Pressure	pancreas, artery aorta, esophagus gastroesophageal junction, artery coronary, artery tibial	Digestive, Cardiovascular
Fasting Glucose	colon sigmoid, adrenal gland, cells EBV-transformed lymphocytes, ovary, heart left ventricle	Digestive, Cardiovascular, Endocrine, Blood/Immune
HDL	cells cultured fibroblasts, brain cerebellum, heart left ventricle, heart atrial appendage, brain cortex	CNS, Cardiovascular
HeelTscore	pancreas, breast mammary tissue, cells EBV-transformed lymphocytes, liver, artery aorta	Digestive, Blood/Immune, Cardiovascular
Height	esophagus gastroesophageal junction, breast mammary tissue, artery aorta, small intestine terminal ileum, adipose subcutaneous	Digestive, Cardiovascular, Blood/Immune
Hypertension	pancreas, liver, artery coronary, artery aorta, breast mammary tissue	Digestive, Cardiovascular
IschemicStrokeAndSubtypes	ovary, cells cultured fibroblasts, minor salivary gland, brain hypothalamus, breast mammary tissue	Endocrine, Digestive, CNS
LDL	liver, pancreas, brain substantia nigra, esophagus mucosa, minor salivary gland	Digestive, CNS
Migraine	artery aorta, artery tibial, spleen, artery coronary, pancreas	Cardiovascular, Digestive, Blood/Immune
Neuroticism	esophagus gastroesophageal junction, breast mammary tissue, esophagus muscularis, brain caudate basal ganglia, liver	Digestive, CNS
Schizophrenia	liver, pancreas, breast mammary tissue, artery aorta, brain cerebellum	Digestive, Cardiovascular, CNS
Smoking Status	liver, esophagus muscularis, brain cerebellum, small intestine terminal ileum, pancreas	Digestive, CNS
Systolic Blood Pressure	pancreas, artery aorta, esophagus gastroesophageal junction, artery coronary, artery tibial	Digestive, Cardiovascular
Triglycerides	cells EBV-transformed lymphocytes, pancreas, prostate, brain hippocampus, skin not sun exposed suprapubic, liver*	Blood/Immune, Digestive, CNS
Years of Education	small intestine terminal ileum, artery aorta, muscle skeletal, cells cultured fibroblasts, brain cerebellum	Digestive, Cardiovascular, CNS

**FIGURE 4:**
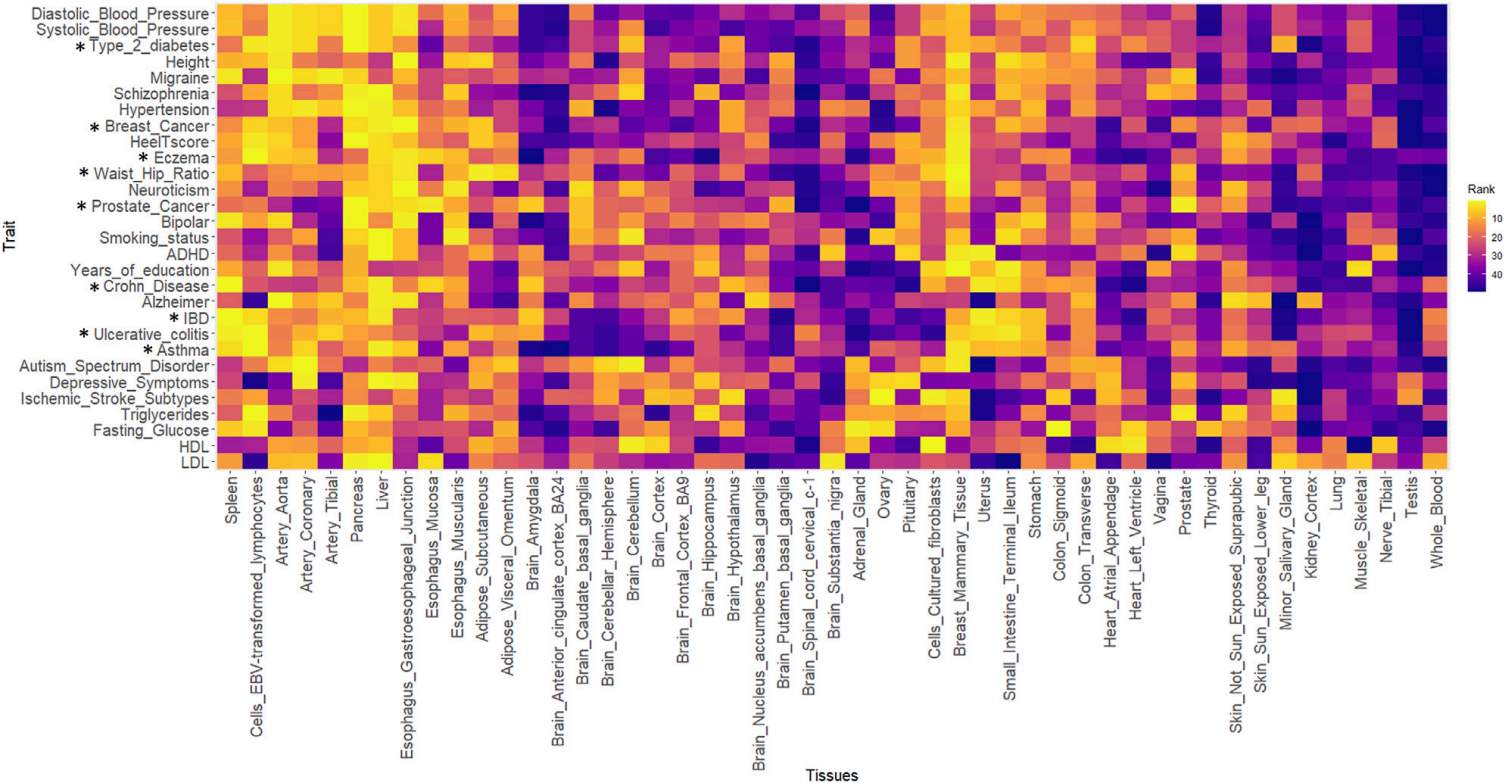
The heatmap for all the traits against 49 GTEx tissues. The yellow colour indicates the tissues that are enriched with regulatory effects for a particular trait. * represents the traits present in the training datasets.

Figure 4 shows the heatmap for all the traits against 49 GTEx tissues. The ranks of the tissues were based on the best-performing method i.e., SZBP. A Pearson correlation matrix was generated using the “cor” function in R (3.6.1). The distance matrix was generated using the Euclidean distance method embedded in the heatmap R package, where the correlation between the rank of the tissues for each trait is used as a distance function (Supplementary Figure S4). Clustering was performed using the “complete linkage method” (Defays, 1977) embedded in the “hclust” function in R. The traits were clustered together and the same re-ordering was used to generate the heatmap in Figure 4. The yellow colour indicates the tissues that are enriched with regulatory effects for a particular trait.

## 4 Discussion

In this study, a novel approach was developed—genome-wide imputed differential expression enrichment (GIDEE)—to prioritise tissues that are enriched for regulatory effects (eQTLs) that are associated with a GWAS trait. This approach was applied to 29 GWAS datasets that were divided into two groups: 1) training datasets and 2) test datasets. The involvement of specific tissues in the pathogenicity of the training datasets had been established and reported in the literature. Therefore, the performance of four enrichment tests and their combinations was benchmarked utilising the training datasets by assessing the GIDEE ranking of the known pathogenic tissue. The best-performing enrichment test combination was utilised in the subsequent GIDEE analysis of the test datasets.

This approach can be viewed as a logical extension of TWAS and the application of tools such as MetaXcan (Barbeira et al., 2018). Although ∼200 studies utilising MetaXcan (Sakornsakolpat et al., 2017; Khawaja et al., 2018; Sanchez-Roige et al., 2019; Guo et al., 2020; Li et al., 2021) and/or other TWAS approaches have been published, less attention has been paid to the quality of genetic predictors/weights. Initial benchmarking for GIDEE using all genes within each tissue resulted in poor prioritisation of expected “known” pathogenic tissues for the training datasets. We suspected that the poor prioritisation was due to random “noise” generated by including poorly performing prediction models in the enrichment tests. Furthermore, it was shown by the authors of TWAS that MetaXcan’s results tend to be more significant as the genetic component of gene expression increases (i.e., larger cross-validated prediction performance R2) (Barbeira et al., 2018).

Employing the hypothesis that enrichment of trait-associated differentially expressed genes should be based on robustly imputed gene expression, genes having a MetaXcan prediction performance R^2^ greater than the median R^2^ for each tissue were taken forward in the GIDEE approach. Of the four enrichment measures examined, the empirical Brown’s method (Poole et al., 2016) performed best individually and performed best overall when combined with the mean squared z-score method. Brown’s method of combining non-independent *p*-values has shown utility for examining gene sets such as in pathway analysis (Devlin et al., 2015; Becher et al., 2018). The implementation of Brown’s method computed the empirical co-variance of *p*-values for each tissue within each trait and used this empirical co-variance to scale the chi-square distribution. The advantage of this approach is that the empirical co-variance calculated is non-parametric (i.e., it does not assume any underlying distribution of *p*-values) and is thus applicable to complex and correlated datasets (Rheinbay et al., 2017).

One study (reported in a bioRxiv preprint) examined TWAS-based differential expression enrichment, which interestingly, used an approach related to our second-best performing mean squared z-score approach to test for tissue enrichment associated with type 2 diabetes (Torres et al., 2017). However, there are two key differences in our mean squared z-score enrichment method. Firstly, our analysis was limited to the top 50th percentile of accurately imputed genes (i.e., prediction performance R^2^ greater than the median R^2^) for each tissue. Secondly, residuals from a linear regression model were used that regressed the mean z-squared value for each tissue on its GTEx tissue sample size to adjust for sample size and quantify tissue enrichment. In contrast, the bioRxiv type 2 diabetes study (Torres et al., 2017) used the mean squared z-score produced by TWAS for all genes and ranked tissues based on the sample size and mean squared z-score. The top tissues having a mean squared z-score rank less than the sample size rank were considered enriched—thus providing only a qualitative (yes/no) enrichment classification of tissues, whereas the GIDEE approach provides a quantitative enrichment measure that provides a ranked prioritisation for each tissue adjusted for GTEx sample size.

To check the stability of the best-performing enrichment test’s (SZBP) ranking across the training datasets, we sequentially dropped datasets having a smaller number of GWAS cases (Supplementary Table S10). The combined mean squared z-score and Brown’s *p*-value (SZBP) consistently remained the top-ranked method after sequentially dropping type 2 diabetes, Crohn’s disease, ulcerative colitis, and eczema.

Although the Brown’s method and mean squared z-score approach performed far better overall than the binomial tests using a Bonferroni-adjusted or FDR significance threshold, the binomial tests were still able to highly rank the known pathogenic tissue for some of the training datasets and implicate tissues belonging to biological systems related to some of the test traits. Given the binomial test approaches utilised the more extreme end of the gene differential expression *p*-value distribution, we expect the binomial tests to be more sensitive to GWAS power and pathogenic tissue homogeneity. That is, given complex traits are not necessarily restricted to a single biological and/or pathogenic pathway limiting enrichment analysis to only genes with tissue-wide significant differential expression will typically result in counts insufficient to provide well-powered binomial tests. Nonetheless, the binomial test enrichment measures can still provide a clear and tangible assessment of tissue enrichment which may assist researchers to prioritise tissue(s), and individual or groups of genes, for follow-up studies. For example, researchers may wish to target specific genes and or tissues based on the strength of their differential expression signals and tissue availability.

The GIDEE approach replicated most of the findings and provided insights into some traits that previous LDSC-SEG GTEx-based analyses did not characterise. Supplementary Table S11 provides a comprehensive list and comparison of the tissues prioritised by GIDEE and LDSC-SEG. Given the LDSC-SEG characterization utilised multiple eQTL and chromatin datasets, whereas GIDEE utilised only the GTEx eQTL data, to provide a direct comparison, we note in the table whether LDSC-SEG was able to prioritise GTEx tissues. Notably, for several traits, GIDEE prioritised tissues using only GTEx data, that LDSC-SEG did not prioritise any tissues for using any gene expression dataset. Reassuringly, many of these novel GIDEE GTEx-based eQTL tissue prioritisations *were* prioritised by LDSC-SEG using chromatin data—e.g., ADHD, depressive symptoms, hypertension, LDL, and migraine.

For ADHD, Alzheimer’s disease, autism spectrum disorder, depressive symptoms, and hypertension, LDSC-SEG did not find any enrichment using gene expression data, but our GIDEE approach was able to implicate endocrine, central nervous system (CNS), vascular, liver, and digestive tissue, respectively—involvement of which is supported by the literature. For the neurological traits ADHD, Alzheimer’s disease, autism spectrum disorder, and depressive symptoms, GIDEE ranked brain tissue in the top 10% of tissues. It is interesting to note that some other tissues such as liver were also highly ranked for Alzheimer’s disease and the association of liver in Alzheimer’s disease has been reported in multiple studies (Nho et al., 2019; Bassendine et al., 2020). Similarly, studies have shown that the risk factors associated with vascular thickening due to accumulation of plaque, are also associated with the progression of Alzheimer’s disease (Kalback et al., 2004). There exists a network of arteries at the base of the brain named the circle of Willis and dysfunction/thickening plays a major role in disease development (Roher et al., 2004). Similarly for ADHD, pituitary tissue was among the top 10% of ranked tissues. The pituitary is the main hormone-producing gland influencing almost all body functions such as growth, blood pressure, and reproduction. There is genetic evidence for the involvement of the hypothalamic-pituitary-adrenal (HPA) axis in ADHD (Ma et al., 2011; Fortier et al., 2013). The HPA axis is a complex set of direct influences and feedback interactions among three main components (hypothalamus, pituitary, and adrenal). It is mainly activated as a response to stress and it is dysregulated in ADHD cases (Raz and Leykin, 2015). Brain and vascular tissue enrichment were found in autism spectrum disorder. Autism is considered a neurological disease (Xiong et al., 2019; Lord et al., 2020); however, some studies also suggest autism is linked with higher blood flow in the white and grey matter of the brain thus suggesting a role for vascular mechanisms in autism (Peterson et al., 2019). A study published in 2016 investigated the post-mortem brains of young patients with autism and indirectly suggested abnormal angiogenesis (Azmitia et al., 2016). Later, in 2020, vascular endothelial impairment was also linked to autism using mice models (Ouellette et al., 2020). GIDEE was not able to find CNS enrichment in the case of bipolar disorder; however, tissues from the digestive system and pancreas were among the top 10% ranked, analogous to some other genetic studies (Finucane et al., 2018). For depressive symptoms and neuroticism, in addition to brain tissues, GIDEE found enrichment for tissues involved in the digestive system (Clapp et al., 2017). For blood pressure traits in the test datasets (i.e., diastolic blood pressure, systolic blood pressure and hypertension) artery tissues were ranked among the top 10% tissues. Lipids have been known to be associated with multiple traits including diseases of the circulatory system such as coronary heart disease (Ference et al., 2018) and diseases of the nervous system such as multiple sclerosis (Reale and Sanchez-Ramon, 2017) and Alzheimer’s disease. Height exhibited differential expression enrichment implicating digestive, vascular, and adipose tissues—in agreement with previous findings (Wood et al., 2014; Finucane et al., 2018). There is an ongoing debate on whether migraine is primarily a disease of neurological or vascular dysfunction. Using GIDEE, vascular tissues were the most strongly enriched for differentially expressed genes, suggesting vascular tissues to be likely pathogenic, in line with previous suggestions (Gormley et al., 2016; Choquet et al., 2021). Lastly, brain cerebellum was ranked among the top 10% tissues for smoking status and years of education—implicating the CNS—as previously reported (Finucane et al., 2018; Xu et al., 2020).

Other important differences between LDSC-SEG and GIDEE include 1) LDSC-SEG eliminates housekeeping and other potentially important trait-related genes that are expressed across multiple tissues; 2) GIDEE assesses gene-tissue enrichment with respect to the gene’s *association* with the GWAS trait; and 3) GIDEE allows for different and tissue-specific regional relationships between GWAS risk SNPs and gene expression (e.g., heterogenous eQTL effect magnitude and direction across tissues).

Two other tissue-prioritisation approaches similar to LDSC-SEG are deTS (Pei et al., 2019) and RolyPoly (Calderon et al., 2017). deTS uses the top 5% of the genes after differential expression analysis using t-statistics and assign this gene list to specific tissues (SEGs). Afterwards, it uses Fisher’s exact test to test for enrichment in focal tissue. deTS was applied to 26 traits and results were similar to GIDEE—i.e., blood and spleen were associated with immune-related traits such as Crohn’s disease, Eczema and Ulcerative Colitis, and brain tissue associated with neurological diseases. RolyPoly, another approach similar to LDSC-SEG, is designed for single-cell expression studies. RolyPoly ranks all genes in a descending order based upon normalised expression values and takes the top 20% of the genes within each tissue as SEGs. Afterwards, RolyPoly creates a binary SNP annotation based on whether a SNP is within a 10 kb window nearby the transcription start site of any SEGs. In the second step, RolyPoly applies the same linear mixed model as used in LDSC-SEG to identify trait-relevant cell types. However, both of these approaches use gene expression levels and eliminated housekeeping genes.

Another method, eQTLEnrich (Gamazon et al., 2018), tests for the enrichment of trait associations among eQTLs in each tissue. For a given trait, eQTLEnrich finds the most significant cis-eQTL per gene in each tissue and extracts GWAS association *p*-values for each set of eQTLs. The *p*-value distribution of each set of eQTL per tissue is then tested for enrichment as compared to an empirical null distribution.

In contrast to the above approaches, GIDEE uses information from tissue-specific genetically regulated expression levels directly related to and associated with the GWAS trait. That is, instead of using a most significant cis-SNP, it aggregates the information from all cis-SNPs that relate to gene expression *via* elastic net regression models. The predicted gene expression is then tested for association with the trait in a tissue-specific manner. It is also important to note that tissues prioritised by GIDEE, in its basic form, means that the trait’s GWAS loci have increased regulatory effects in the tissue that are associated with the trait. Such enrichment may be due to the tissue being pathogenic, or because the tissue has increased co-regulatory effects with a pathogenic tissue. Importantly, our GIDEE approach is based on existing and well-characterised TWAS methods but uses TWAS results in a new and creative way.

GIDEE utilises the GTEx data set for the prioritisation of tissues relevant to the trait’s regulatory architecture. GTEx is the most comprehensive transcriptome dataset collected from multiple tissue samples from nearly 1000 individuals and sequenced to high coverage. It provides a comprehensive cross-tissue survey of the functional consequences of genetic variation at the transcript level (Barbeira et al., 2018). Introducing additional eQTL datasets on reduced and heterogeneous subsets of tissues will introduce selection bias into the TWAS results and their subsequent comparison for the enrichment of regulatory effects at GWAS loci.

One potential limitation of GIDEE is that it outputs the prioritisation (ranking) of the trait-associated tissues and not a formal statistical test comparing tissues. However, we note that our approach’s rankings are based on relevant and sound statistical enrichment tests and that these enrichment tests rely upon the results generated by TWAS which has been shown to have a robust type 1 error rate. We also limited the enrichment tests to gene-trait associations with higher prediction performance R2 and the average z-score and Brown’s test produce valid estimates of enrichment of differentially expressed genes on which the trait-associated tissues are ranked. It is difficult to envisage a formal statistical test comparing enrichment across tissues. Issues that would complicate a more formal test include differences in sample sizes and heterogenous lists of differentially expressed genes across tissues.

It is interesting to note that some tissues which appear not to be obviously related and relevant to some traits were prioritised by GIDEE. For example, breast mammary tissue in ADHD, autism spectrum disorder, hypertension, ischemic stroke, neuroticism, and schizophrenia. We hypothesise that such tissue prioritisations could result from individual or combinations of factors such as GTEx tissue sample sizes, gene co-regulation/co-expression (e.g., between a prioritised tissue and a true pathogenic tissue), and/or isoform abundance of pathogenic genes (i.e., GIDEE currently tests for enrichment of differential *total* gene expression; however particular isoforms associated with a trait may be more abundant in non-obvious prioritised tissues). Moreover, gene regulation mechanisms are complicated by context specificity, feedback loops, and hidden confounders in expression data. To further elaborate on this point, we also measured the correlation in gene expression across the 49 GTEx tissues and found that breast mammary tissue had a high gene expression correlation (*r* > 0.9) with all 13 brain tissues present in GTEx. This may explain why breast mammary tissue is being highlighted in multiple neurological traits (i.e., due to expression correlation in the larger GTEx breast mammary tissue which has a larger sample size compared to the brain tissues). Therefore, although the regulatory mechanism may be shared across tissues (Ward et al., 2015), an agnostic scanning of multiple tissues provides us with an additional window of opportunity to detect relevant regulatory activity and develop potential proxy tissue/cell models.

Our results show that the application of our GIDEE approach to GWAS summary statistics can provide important prioritisation of putative pathogenic tissues and/or accessible proxy tissues that will aid in the design of follow-up laboratory studies aimed at functionally characterising GWAS risk loci.

## URLs

https://github.com/AmmarahGhaffar/GIDEE.git

## Data Availability

The original contributions presented in the study are publicly available. This data can be found here: https://github.com/AmmarahGhaffar/GIDEE.git.
