# Supplementary material for: Genome-wide imputed differential expression enrichment analysis identifies trait-relevant tissues
Source: Front Genet. 2023 Jan 6;13:1008511. doi: 10.3389/fgene.2022.1008511 (PMC9870027; doi:10.3389/fgene.2022.1008511)
Supplement: Supplementary file 1 [file DataSheet2.PDF]

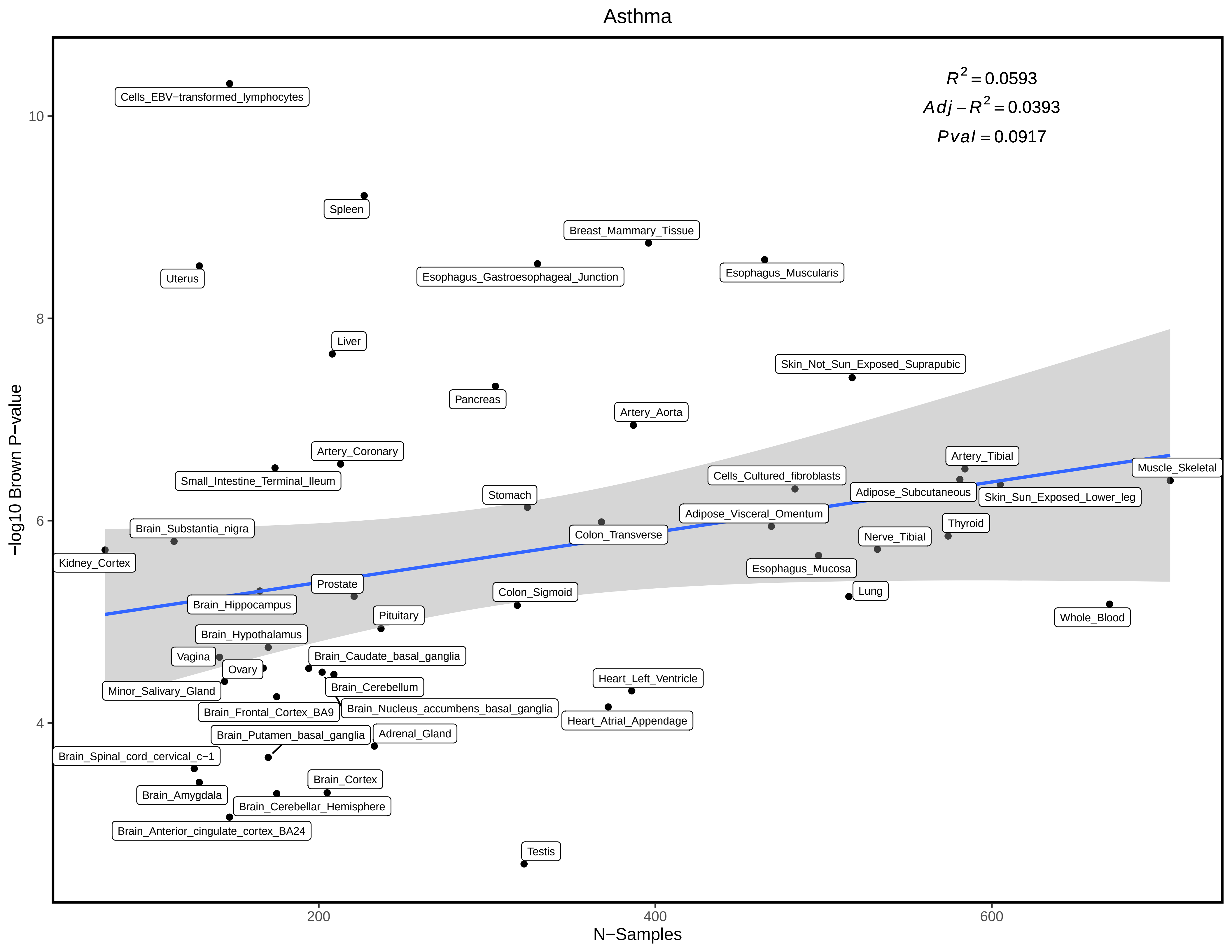

Asthma
$R^2 = 0.0593$
$Adj - R^2 = 0.0393$
$Pval = 0.0917$
−log10 Brown P−value
N−Samples
10
8
6
4
200
400
600
Cells_EBV−transformed_lymphocytes
Spleen
Breast_Mammary_Tissue
Uterus
Esophagus_Gastroesophageal_Junction
Esophagus_Muscularis
Liver
Skin_Not_Sun_Exposed_Suprapubic
Pancreas
Artery_Aorta
Artery_Coronary
Small_Intestine_Terminal_Ileum
Artery_Tibial
Cells_Cultured_fibroblasts
Muscle_Skeletal
Stomach
Adipose_Subcutaneous
Skin_Sun_Exposed_Lower_leg
Adipose_Visceral_Omentum
Thyroid
Brain_Substantia_nigra
Colon_Transverse
Nerve_Tibial
Kidney_Cortex
Esophagus_Mucosa
Prostate
Colon_Sigmoid
Lung
Brain_Hippocampus
Whole_Blood
Pituitary
Brain_Hypothalamus
Vagina
Brain_Caudate_basal_ganglia
Ovary
Heart_Left_Ventricle
Minor_Salivary_Gland
Brain_Cerebellum
Brain_Nucleus_accumbens_basal_ganglia
Brain_Frontal_Cortex_BA9
Heart_Atrial_Appendage
Brain_Putamen_basal_ganglia
Adrenal_Gland
Brain_Spinal_cord_cervical_c−1
Brain_Cortex
Brain_Amygdala
Brain_Cerebellar_Hemisphere
Brain_Anterior_cingulate_cortex_BA24
Testis

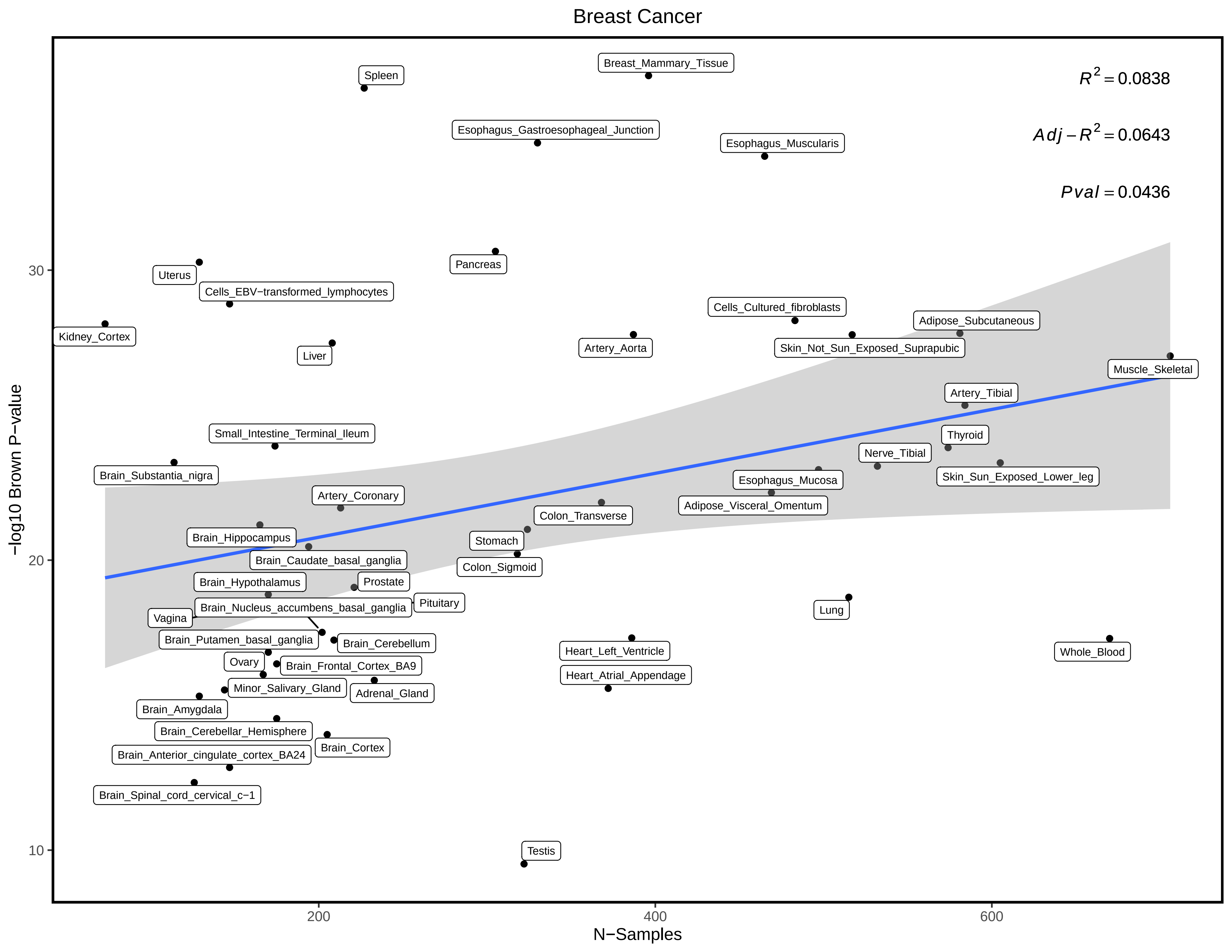

Breast Cancer
$R^2 = 0.0838$
$Adj-R^2 = 0.0643$
$Pval = 0.0436$
−log10 Brown P−value
N−Samples
10
20
30
200
400
600
Spleen
Breast_Mammary_Tissue
Esophagus_Gastroesophageal_Junction
Esophagus_Muscularis
Pancreas
Uterus
Cells_EBV−transformed_lymphocytes
Kidney_Cortex
Liver
Artery_Aorta
Cells_Cultured_fibroblasts
Adipose_Subcutaneous
Skin_Not_Sun_Exposed_Suprapubic
Muscle_Skeletal
Artery_Tibial
Small_Intestine_Terminal_Ileum
Thyroid
Nerve_Tibial
Brain_Substantia_nigra
Skin_Sun_Exposed_Lower_leg
Esophagus_Mucosa
Artery_Coronary
Adipose_Visceral_Omentum
Colon_Transverse
Brain_Hippocampus
Stomach
Brain_Caudate_basal_ganglia
Colon_Sigmoid
Brain_Hypothalamus
Prostate
Pituitary
Brain_Nucleus_accumbens_basal_ganglia
Vagina
Lung
Brain_Putamen_basal_ganglia
Brain_Cerebellum
Heart_Left_Ventricle
Whole_Blood
Ovary
Brain_Frontal_Cortex_BA9
Heart_Atrial_Appendage
Minor_Salivary_Gland
Adrenal_Gland
Brain_Amygdala
Brain_Cerebellar_Hemisphere
Brain_Cortex
Brain_Anterior_cingulate_cortex_BA24
Brain_Spinal_cord_cervical_c−1
Testis

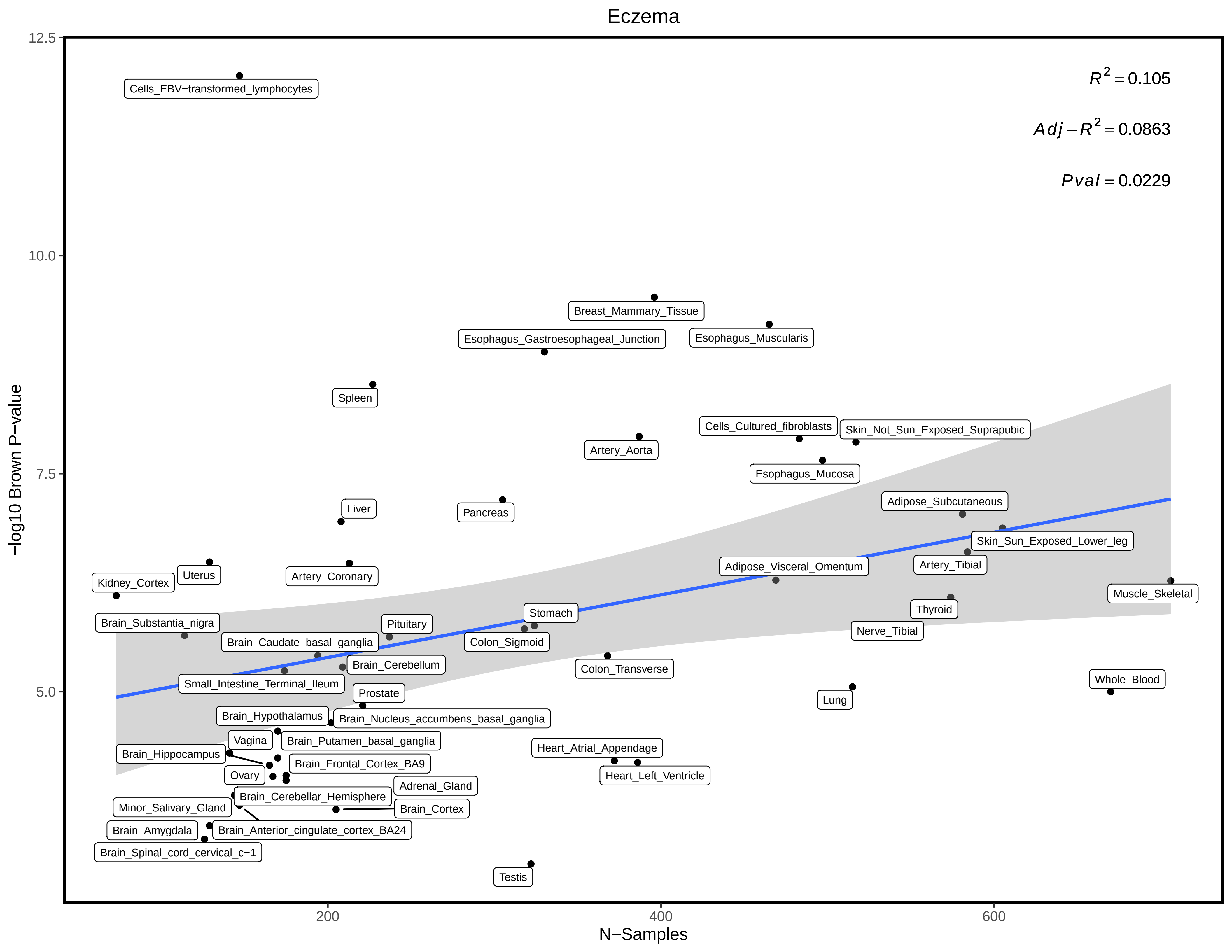

Eczema
$R^2 = 0.105$
$Adj - R^2 = 0.0863$
$Pval = 0.0229$
−log10 Brown P−value
N−Samples
12.5
10.0
7.5
5.0
200
400
600
Cells_EBV−transformed_lymphocytes
Breast_Mammary_Tissue
Esophagus_Gastroesophageal_Junction
Esophagus_Muscularis
Spleen
Cells_Cultured_fibroblasts
Skin_Not_Sun_Exposed_Suprapubic
Artery_Aorta
Esophagus_Mucosa
Liver
Pancreas
Adipose_Subcutaneous
Skin_Sun_Exposed_Lower_leg
Uterus
Artery_Coronary
Kidney_Cortex
Adipose_Visceral_Omentum
Artery_Tibial
Muscle_Skeletal
Thyroid
Stomach
Brain_Substantia_nigra
Pituitary
Nerve_Tibial
Brain_Caudate_basal_ganglia
Colon_Sigmoid
Brain_Cerebellum
Colon_Transverse
Small_Intestine_Terminal_Ileum
Whole_Blood
Prostate
Lung
Brain_Hypothalamus
Brain_Nucleus_accumbens_basal_ganglia
Vagina
Brain_Putamen_basal_ganglia
Heart_Atrial_Appendage
Brain_Hippocampus
Brain_Frontal_Cortex_BA9
Ovary
Heart_Left_Ventricle
Adrenal_Gland
Brain_Cerebellar_Hemisphere
Minor_Salivary_Gland
Brain_Cortex
Brain_Amygdala
Brain_Anterior_cingulate_cortex_BA24
Brain_Spinal_cord_cervical_c−1
Testis

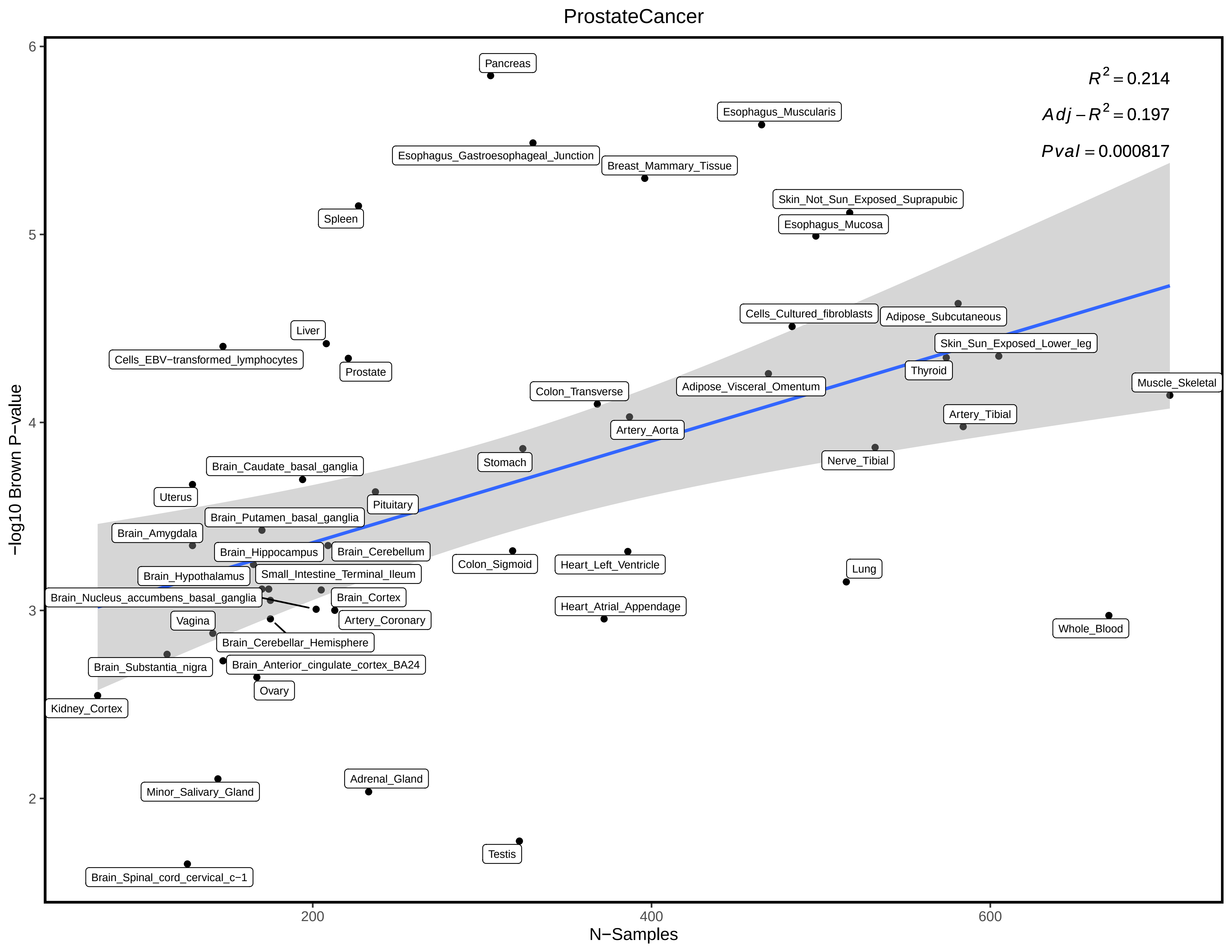

ProstateCancer
$R^2 = 0.214$
$Adj - R^2 = 0.197$
$Pval = 0.000817$
-log10 Brown P-value
N-Samples
6
5
4
3
2
200
400
600
Pancreas
Esophagus_Muscularis
Esophagus_Gastroesophageal_Junction
Breast_Mammary_Tissue
Skin_Not_Sun_Exposed_Suprapubic
Spleen
Esophagus_Mucosa
Cells_Cultured_fibroblasts
Adipose_Subcutaneous
Liver
Cells_EBV-transformed_lymphocytes
Prostate
Skin_Sun_Exposed_Lower_leg
Thyroid
Adipose_Visceral_Omentum
Colon_Transverse
Muscle_Skeletal
Artery_Tibial
Artery_Aorta
Stomach
Nerve_Tibial
Brain_Caudate_basal_ganglia
Uterus
Pituitary
Brain_Putamen_basal_ganglia
Brain_Amygdala
Brain_Hippocampus
Brain_Cerebellum
Colon_Sigmoid
Heart_Left_Ventricle
Lung
Brain_Hypothalamus
Small_Intestine_Terminal_Ileum
Brain_Nucleus_accumbens_basal_ganglia
Brain_Cortex
Heart_Atrial_Appendage
Vagina
Artery_Coronary
Whole_Blood
Brain_Cerebellar_Hemisphere
Brain_Substantia_nigra
Brain_Anterior_cingulate_cortex_BA24
Ovary
Kidney_Cortex
Adrenal_Gland
Minor_Salivary_Gland
Testis
Brain_Spinal_cord_cervical_c-1

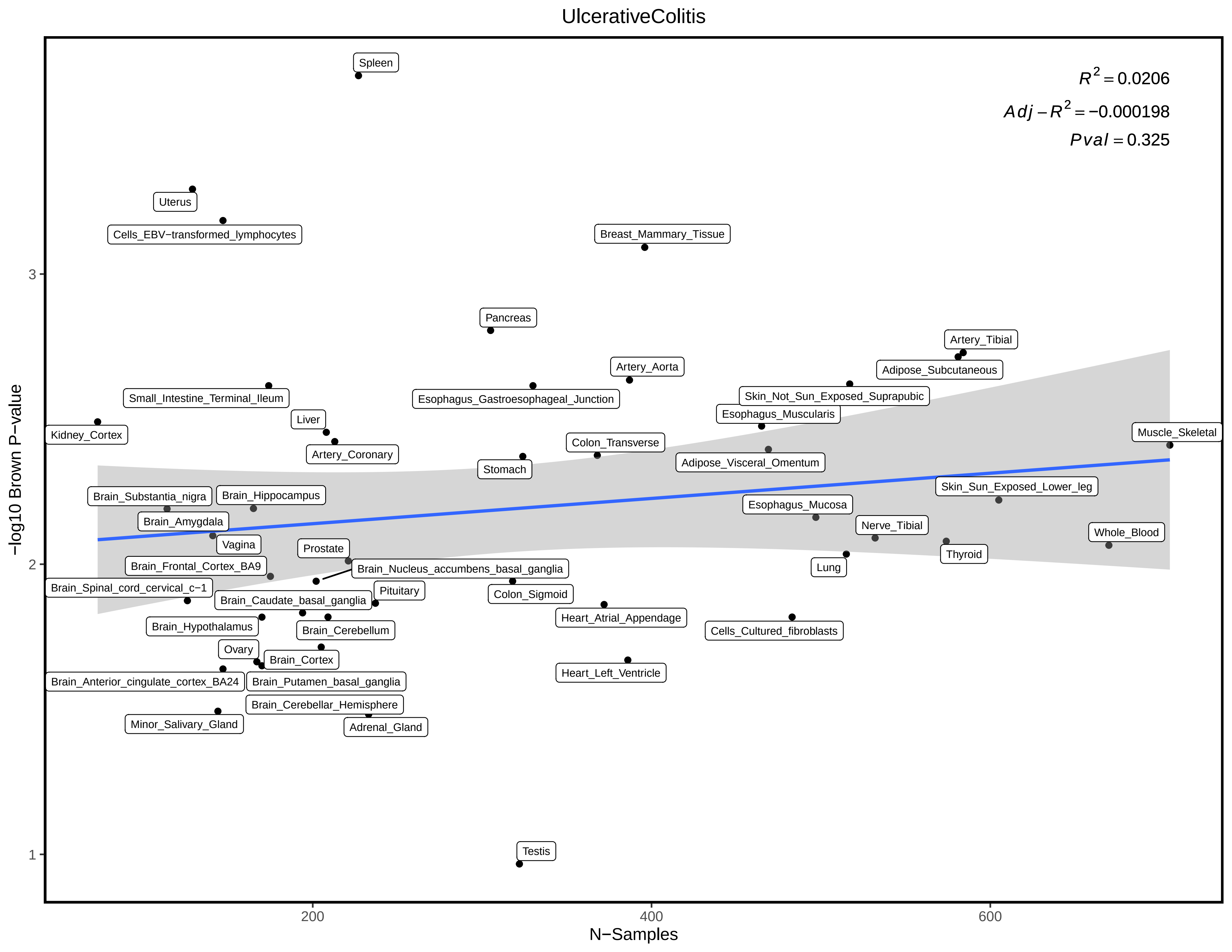
UlcerativeColitis
$R^2 = 0.0206$
$Adj-R^2 = -0.000198$
$Pval = 0.325$
−log10 Brown P−value
N−Samples
Spleen
Uterus
Cells_EBV−transformed_lymphocytes
Breast_Mammary_Tissue
Pancreas
Artery_Tibial
Adipose_Subcutaneous
Artery_Aorta
Small_Intestine_Terminal_Ileum
Esophagus_Gastroesophageal_Junction
Skin_Not_Sun_Exposed_Suprapubic
Esophagus_Muscularis
Liver
Kidney_Cortex
Artery_Coronary
Colon_Transverse
Muscle_Skeletal
Stomach
Adipose_Visceral_Omentum
Skin_Sun_Exposed_Lower_leg
Brain_Substantia_nigra
Brain_Hippocampus
Esophagus_Mucosa
Brain_Amygdala
Nerve_Tibial
Whole_Blood
Vagina
Prostate
Thyroid
Brain_Frontal_Cortex_BA9
Lung
Brain_Nucleus_accumbens_basal_ganglia
Brain_Spinal_cord_cervical_c−1
Pituitary
Colon_Sigmoid
Brain_Caudate_basal_ganglia
Brain_Hypothalamus
Heart_Atrial_Appendage
Brain_Cerebellum
Cells_Cultured_fibroblasts
Ovary
Brain_Cortex
Heart_Left_Ventricle
Brain_Anterior_cingulate_cortex_BA24
Brain_Putamen_basal_ganglia
Brain_Cerebellar_Hemisphere
Minor_Salivary_Gland
Adrenal_Gland
Testis
1
2
3
200
400
600

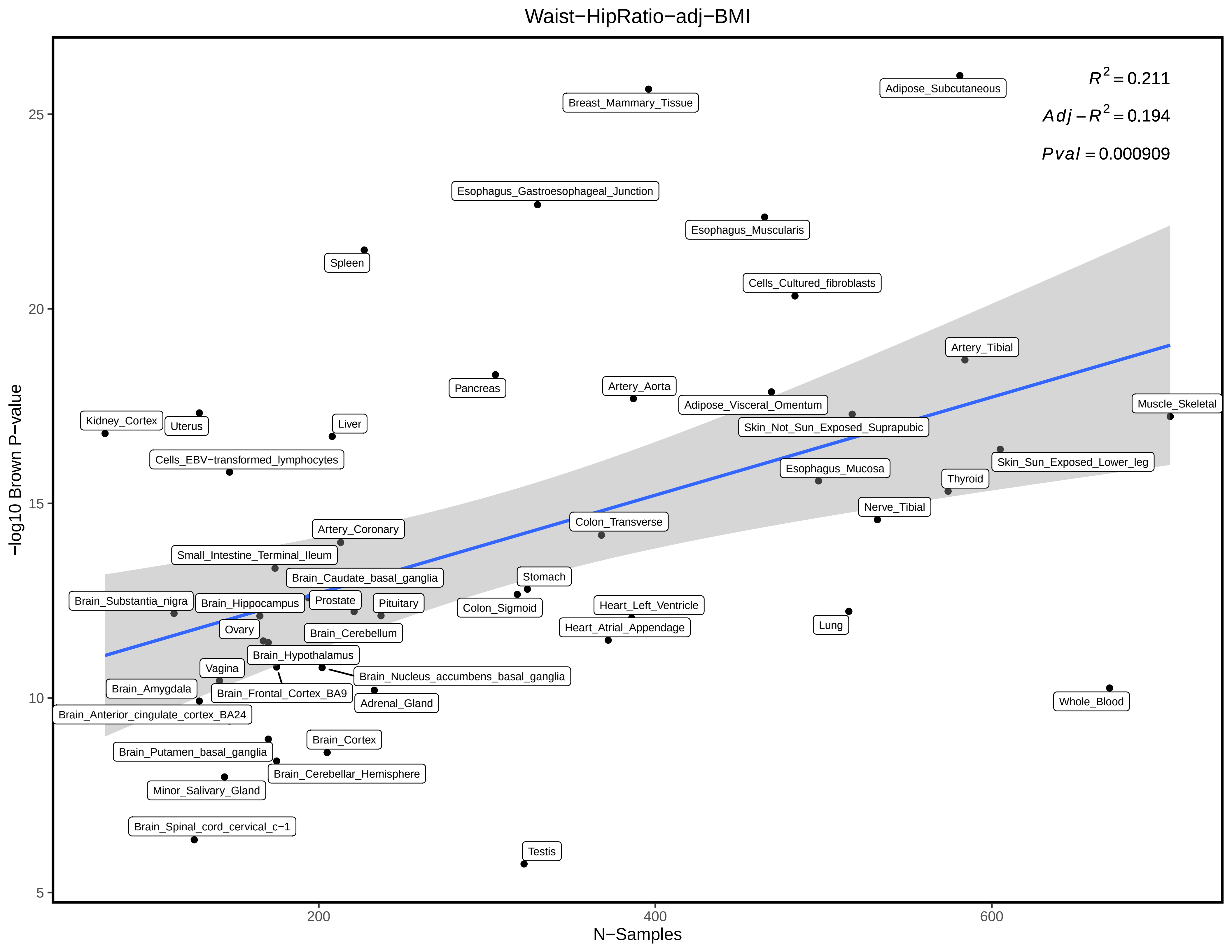
Waist−HipRatio−adj−BMI
$R^2 = 0.211$
$Adj - R^2 = 0.194$
$Pval = 0.000909$
−log10 Brown P−value
N−Samples
25
20
15
10
5
200
400
600
Adipose_Subcutaneous
Breast_Mammary_Tissue
Esophagus_Gastroesophageal_Junction
Esophagus_Muscularis
Spleen
Cells_Cultured_fibroblasts
Artery_Tibial
Pancreas
Artery_Aorta
Adipose_Visceral_Omentum
Muscle_Skeletal
Kidney_Cortex
Uterus
Liver
Skin_Not_Sun_Exposed_Suprapubic
Cells_EBV−transformed_lymphocytes
Skin_Sun_Exposed_Lower_leg
Esophagus_Mucosa
Thyroid
Nerve_Tibial
Colon_Transverse
Artery_Coronary
Small_Intestine_Terminal_Ileum
Brain_Caudate_basal_ganglia
Stomach
Brain_Substantia_nigra
Brain_Hippocampus
Prostate
Pituitary
Colon_Sigmoid
Heart_Left_Ventricle
Heart_Atrial_Appendage
Lung
Ovary
Brain_Cerebellum
Brain_Hypothalamus
Vagina
Brain_Nucleus_accumbens_basal_ganglia
Brain_Amygdala
Brain_Frontal_Cortex_BA9
Adrenal_Gland
Whole_Blood
Brain_Anterior_cingulate_cortex_BA24
Brain_Cortex
Brain_Putamen_basal_ganglia
Brain_Cerebellar_Hemisphere
Minor_Salivary_Gland
Brain_Spinal_cord_cervical_c−1
Testis

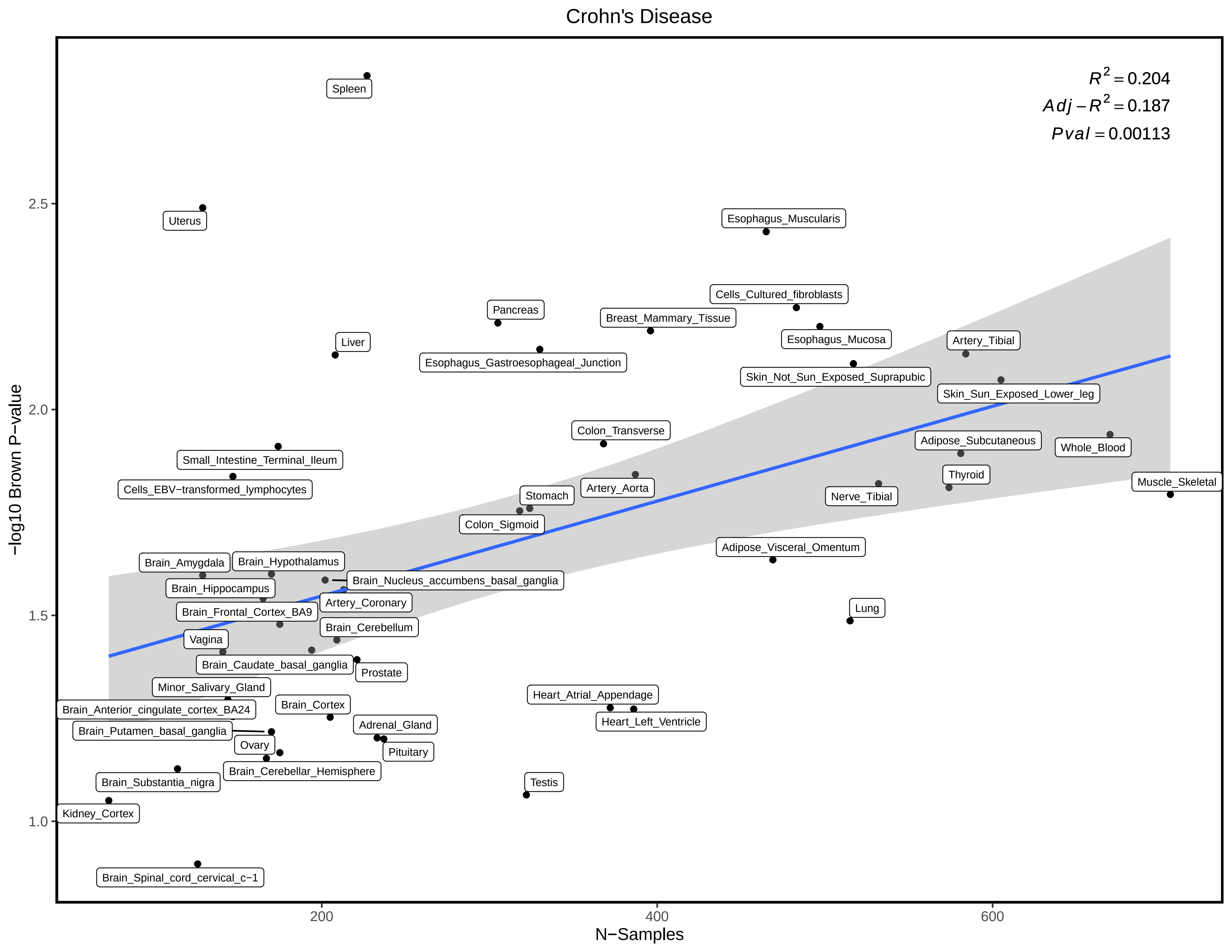

Crohn's Disease
$R^2 = 0.204$
$Adj - R^2 = 0.187$
$Pval = 0.00113$
−log10 Brown P−value
N−Samples
2.5
2.0
1.5
1.0
200
400
600
Spleen
Uterus
Esophagus_Muscularis
Cells_Cultured_fibroblasts
Pancreas
Breast_Mammary_Tissue
Liver
Esophagus_Mucosa
Artery_Tibial
Esophagus_Gastroesophageal_Junction
Skin_Not_Sun_Exposed_Suprapubic
Skin_Sun_Exposed_Lower_leg
Colon_Transverse
Adipose_Subcutaneous
Whole_Blood
Small_Intestine_Terminal_Ileum
Thyroid
Muscle_Skeletal
Cells_EBV−transformed_lymphocytes
Stomach
Artery_Aorta
Nerve_Tibial
Colon_Sigmoid
Adipose_Visceral_Omentum
Brain_Amygdala
Brain_Hypothalamus
Brain_Nucleus_accumbens_basal_ganglia
Brain_Hippocampus
Artery_Coronary
Lung
Brain_Frontal_Cortex_BA9
Brain_Cerebellum
Vagina
Brain_Caudate_basal_ganglia
Prostate
Minor_Salivary_Gland
Heart_Atrial_Appendage
Brain_Cortex
Brain_Anterior_cingulate_cortex_BA24
Heart_Left_Ventricle
Adrenal_Gland
Brain_Putamen_basal_ganglia
Ovary
Pituitary
Brain_Cerebellar_Hemisphere
Brain_Substantia_nigra
Testis
Kidney_Cortex
Brain_Spinal_cord_cervical_c−1

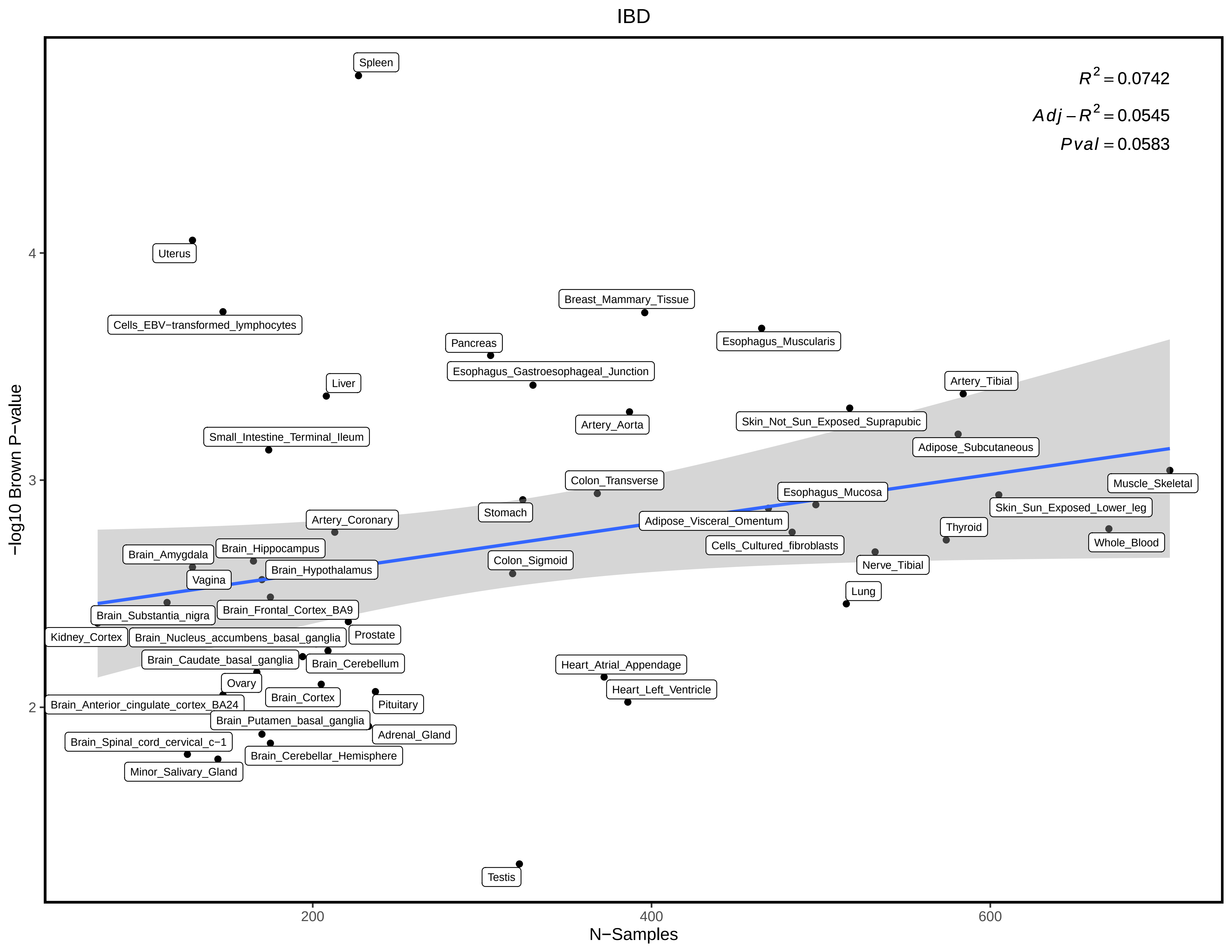

IBD
$R^2 = 0.0742$
$Adj - R^2 = 0.0545$
$Pval = 0.0583$
-log10 Brown P-value
N-Samples
4
3
2
200
400
600
Spleen
Uterus
Cells_EBV-transformed_lymphocytes
Breast_Mammary_Tissue
Esophagus_Muscularis
Pancreas
Esophagus_Gastroesophageal_Junction
Liver
Artery_Tibial
Artery_Aorta
Skin_Not_Sun_Exposed_Suprapubic
Small_Intestine_Terminal_Ileum
Adipose_Subcutaneous
Colon_Transverse
Muscle_Skeletal
Esophagus_Mucosa
Stomach
Skin_Sun_Exposed_Lower_leg
Artery_Coronary
Adipose_Visceral_Omentum
Thyroid
Brain_Hippocampus
Cells_Cultured_fibroblasts
Whole_Blood
Brain_Amygdala
Colon_Sigmoid
Nerve_Tibial
Brain_Hypothalamus
Vagina
Lung
Brain_Frontal_Cortex_BA9
Brain_Substantia_nigra
Kidney_Cortex
Brain_Nucleus_accumbens_basal_ganglia
Prostate
Brain_Caudate_basal_ganglia
Brain_Cerebellum
Heart_Atrial_Appendage
Ovary
Heart_Left_Ventricle
Brain_Cortex
Brain_Anterior_cingulate_cortex_BA24
Pituitary
Brain_Putamen_basal_ganglia
Adrenal_Gland
Brain_Spinal_cord_cervical_c-1
Brain_Cerebellar_Hemisphere
Minor_Salivary_Gland
Testis

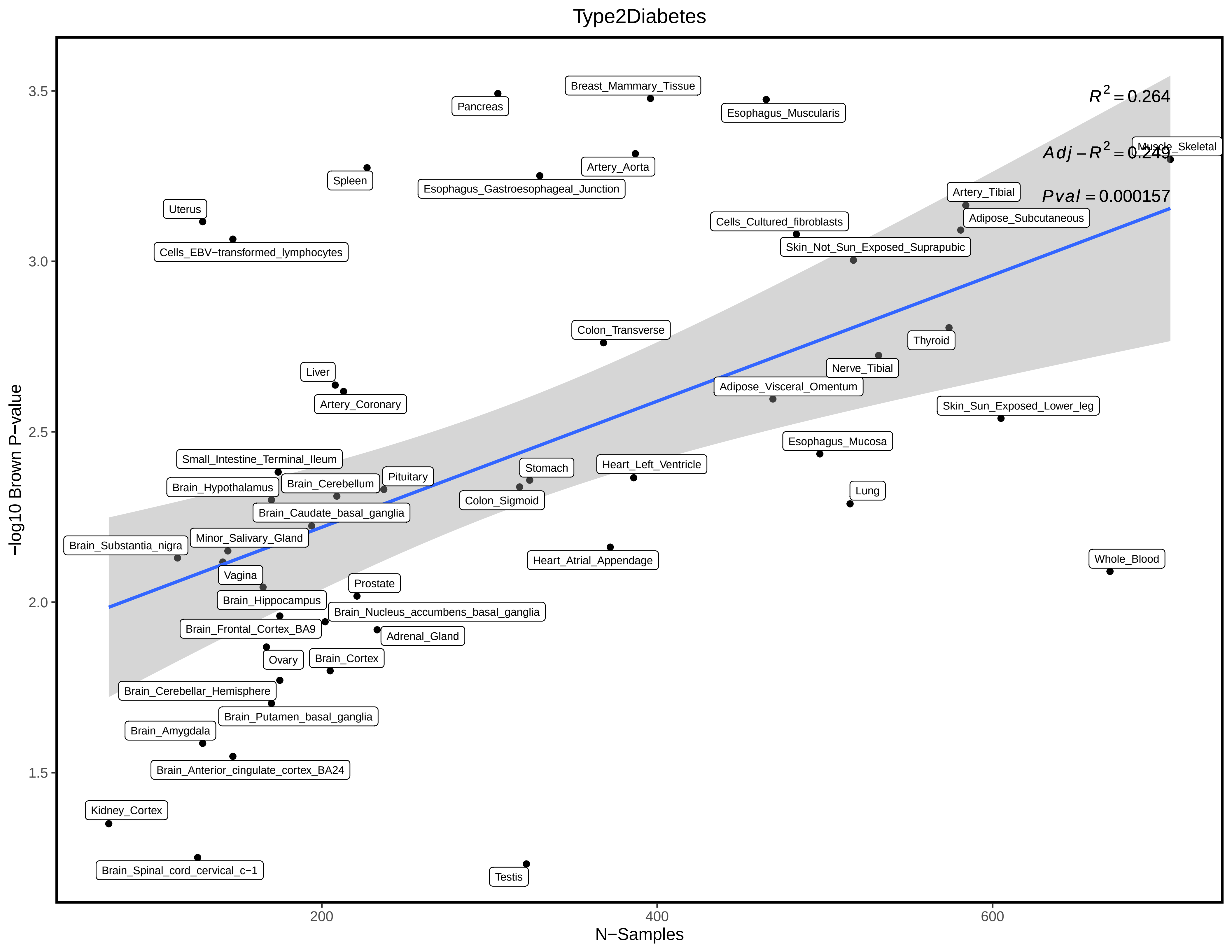

Type2Diabetes
$R^2 = 0.264$
$Adj - R^2 = 0.249$
$Pval = 0.000157$
-log10 Brown P-value
N-Samples
3.5
3.0
2.5
2.0
1.5
200
400
600
Pancreas
Breast_Mammary_Tissue
Esophagus_Muscularis
Muscle_Skeletal
Artery_Aorta
Spleen
Esophagus_Gastroesophageal_Junction
Artery_Tibial
Uterus
Adipose_Subcutaneous
Cells_Cultured_fibroblasts
Cells_EBV-transformed_lymphocytes
Skin_Not_Sun_Exposed_Suprapubic
Colon_Transverse
Thyroid
Nerve_Tibial
Liver
Adipose_Visceral_Omentum
Artery_Coronary
Skin_Sun_Exposed_Lower_leg
Esophagus_Mucosa
Small_Intestine_Terminal_Ileum
Stomach
Heart_Left_Ventricle
Pituitary
Brain_Cerebellum
Brain_Hypothalamus
Colon_Sigmoid
Lung
Brain_Caudate_basal_ganglia
Minor_Salivary_Gland
Brain_Substantia_nigra
Heart_Atrial_Appendage
Whole_Blood
Vagina
Prostate
Brain_Hippocampus
Brain_Nucleus_accumbens_basal_ganglia
Brain_Frontal_Cortex_BA9
Adrenal_Gland
Ovary
Brain_Cortex
Brain_Cerebellar_Hemisphere
Brain_Putamen_basal_ganglia
Brain_Amygdala
Brain_Anterior_cingulate_cortex_BA24
Kidney_Cortex
Brain_Spinal_cord_cervical_c-1
Testis
