# Supplementary figures and images for: Genome-wide imputed differential expression enrichment analysis identifies trait-relevant tissues
Source: Front Genet. 2023 Jan 6;13:1008511. doi: 10.3389/fgene.2022.1008511 (PMC9870027; doi:10.3389/fgene.2022.1008511)

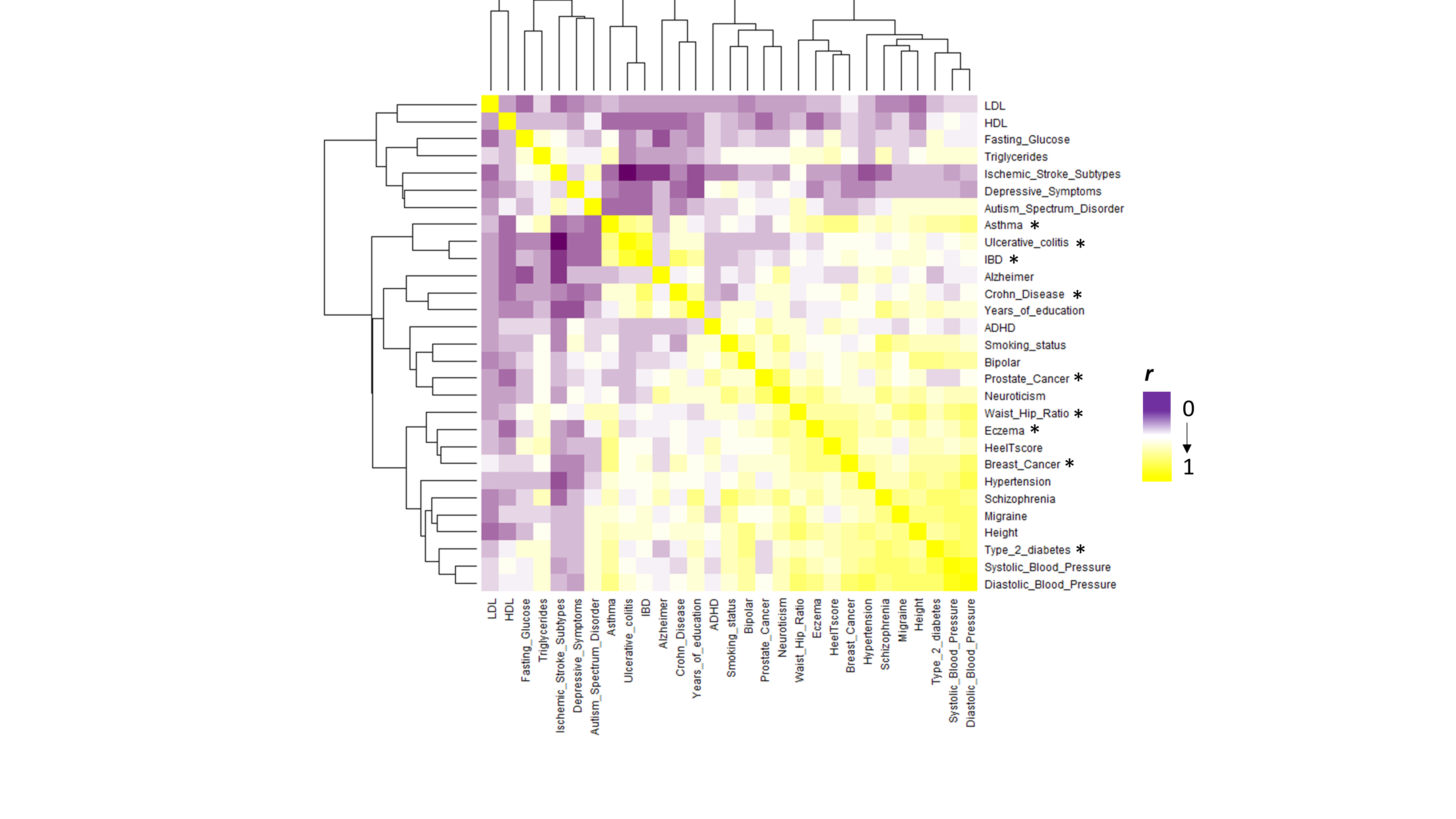

Supplement: Supplementary file 5 [file Image1.TIF]
