# Supplementary material for: Genome-wide imputed differential expression enrichment analysis identifies trait-relevant tissues
Source: Front Genet. 2023 Jan 6;13:1008511. doi: 10.3389/fgene.2022.1008511 (PMC9870027; doi:10.3389/fgene.2022.1008511)
Supplement: Supplementary file 6 [file DataSheet3.pdf]

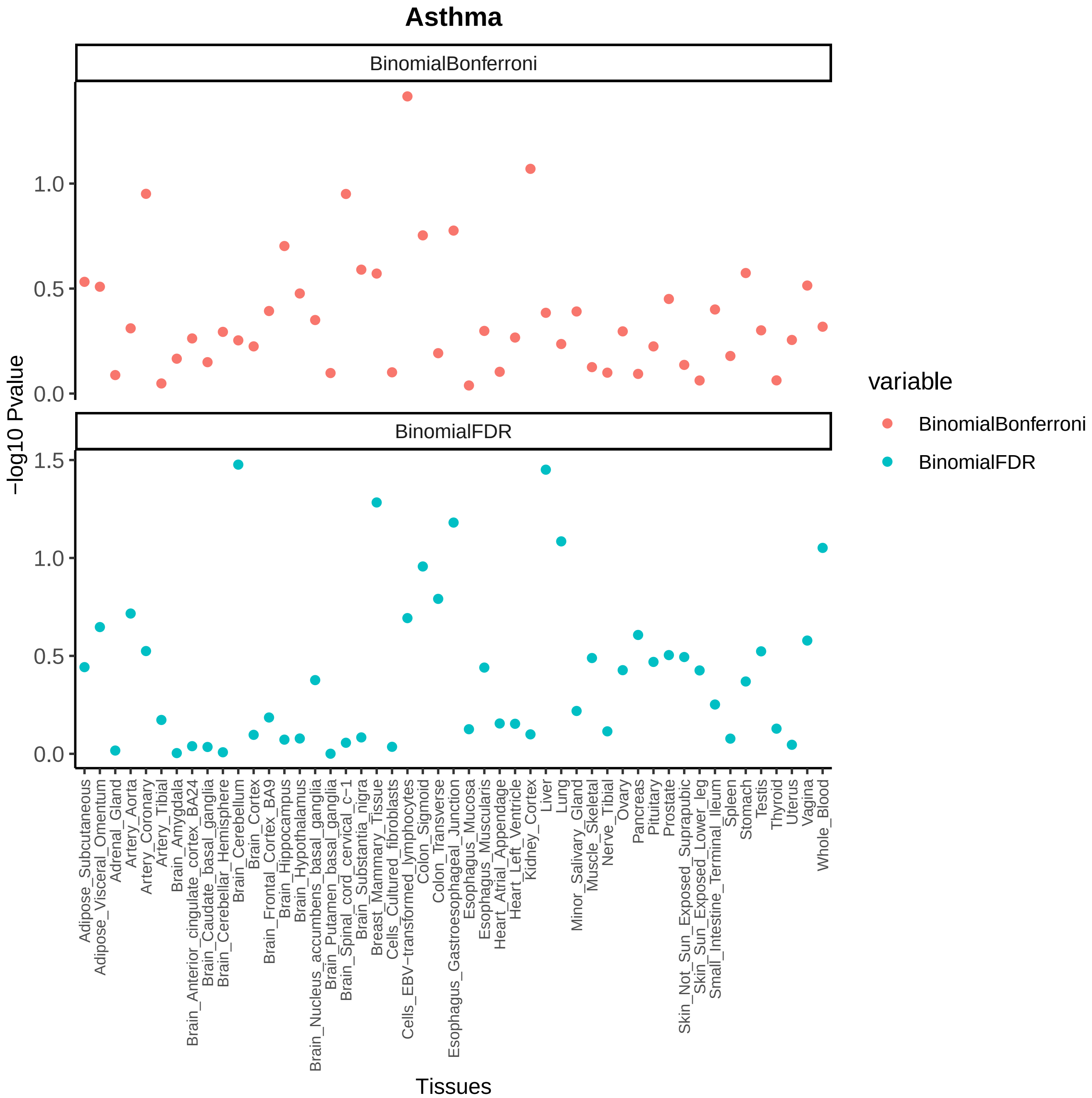

Asthma
BinomialBonferroni
BinomialFDR
−log10 Pvalue
0.0
0.5
1.0
1.5
Tissues
variable
BinomialBonferroni
BinomialFDR
Adipose_Subcutaneous
Adipose_Visceral_Omentum
Adrenal_Gland
Artery_Aorta
Artery_Coronary
Artery_Tibial
Brain_Amygdala
Brain_Anterior_cingulate_cortex_BA24
Brain_Caudate_basal_ganglia
Brain_Cerebellar_Hemisphere
Brain_Cerebellum
Brain_Cortex
Brain_Frontal_Cortex_BA9
Brain_Hippocampus
Brain_Hypothalamus
Brain_Nucleus_accumbens_basal_ganglia
Brain_Putamen_basal_ganglia
Brain_Spinal_cord_cervical_c−1
Brain_Substantia_nigra
Breast_Mammary_Tissue
Cells_Cultured_fibroblasts
Cells_EBV−transformed_lymphocytes
Colon_Sigmoid
Colon_Transverse
Esophagus_Gastroesophageal_Junction
Esophagus_Mucosa
Esophagus_Muscularis
Heart_Atrial_Appendage
Heart_Left_Ventricle
Kidney_Cortex
Liver
Lung
Minor_Salivary_Gland
Muscle_Skeletal
Nerve_Tibial
Ovary
Pancreas
Pituitary
Prostate
Skin_Not_Sun_Exposed_Suprapubic
Skin_Sun_Exposed_Lower_leg
Small_Intestine_Terminal_Ileum
Spleen
Stomach
Testis
Thyroid
Uterus
Vagina
Whole_Blood

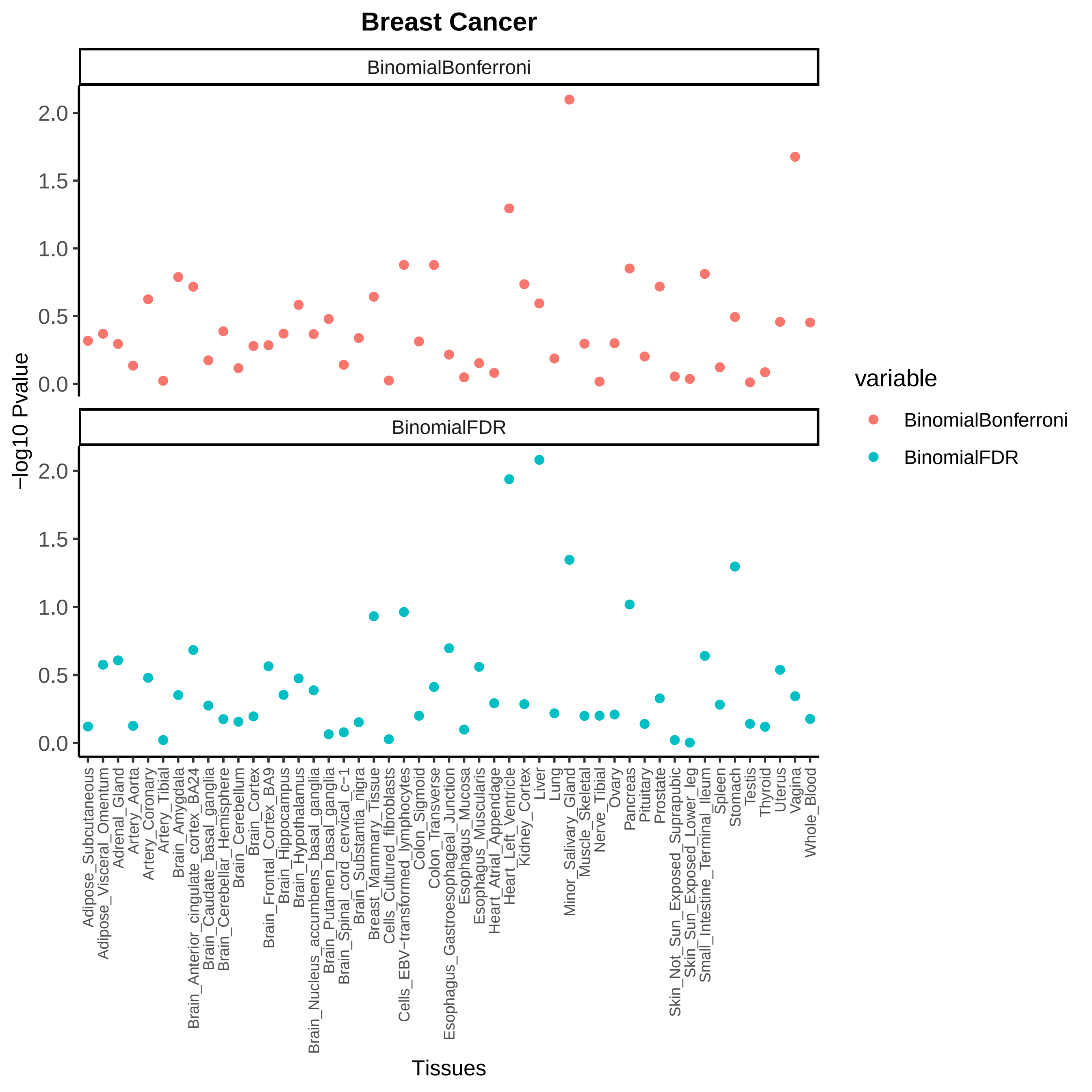
Breast Cancer
BinomialBonferroni
BinomialFDR
−log10 Pvalue
Tissues
variable
BinomialBonferroni
BinomialFDR
0.0
0.5
1.0
1.5
2.0
Adipose_Subcutaneous
Adipose_Visceral_Omentum
Adrenal_Gland
Artery_Aorta
Artery_Coronary
Artery_Tibial
Brain_Amygdala
Brain_Anterior_cingulate_cortex_BA24
Brain_Caudate_basal_ganglia
Brain_Cerebellar_Hemisphere
Brain_Cerebellum
Brain_Cortex
Brain_Frontal_Cortex_BA9
Brain_Hippocampus
Brain_Hypothalamus
Brain_Nucleus_accumbens_basal_ganglia
Brain_Putamen_basal_ganglia
Brain_Spinal_cord_cervical_c−1
Brain_Substantia_nigra
Breast_Mammary_Tissue
Cells_Cultured_fibroblasts
Cells_EBV−transformed_lymphocytes
Colon_Sigmoid
Colon_Transverse
Esophagus_Gastroesophageal_Junction
Esophagus_Mucosa
Esophagus_Muscularis
Heart_Atrial_Appendage
Heart_Left_Ventricle
Kidney_Cortex
Liver
Lung
Minor_Salivary_Gland
Muscle_Skeletal
Nerve_Tibial
Ovary
Pancreas
Pituitary
Prostate
Skin_Not_Sun_Exposed_Suprapubic
Skin_Sun_Exposed_Lower_leg
Small_Intestine_Terminal_Ileum
Spleen
Stomach
Testis
Thyroid
Uterus
Vagina
Whole_Blood

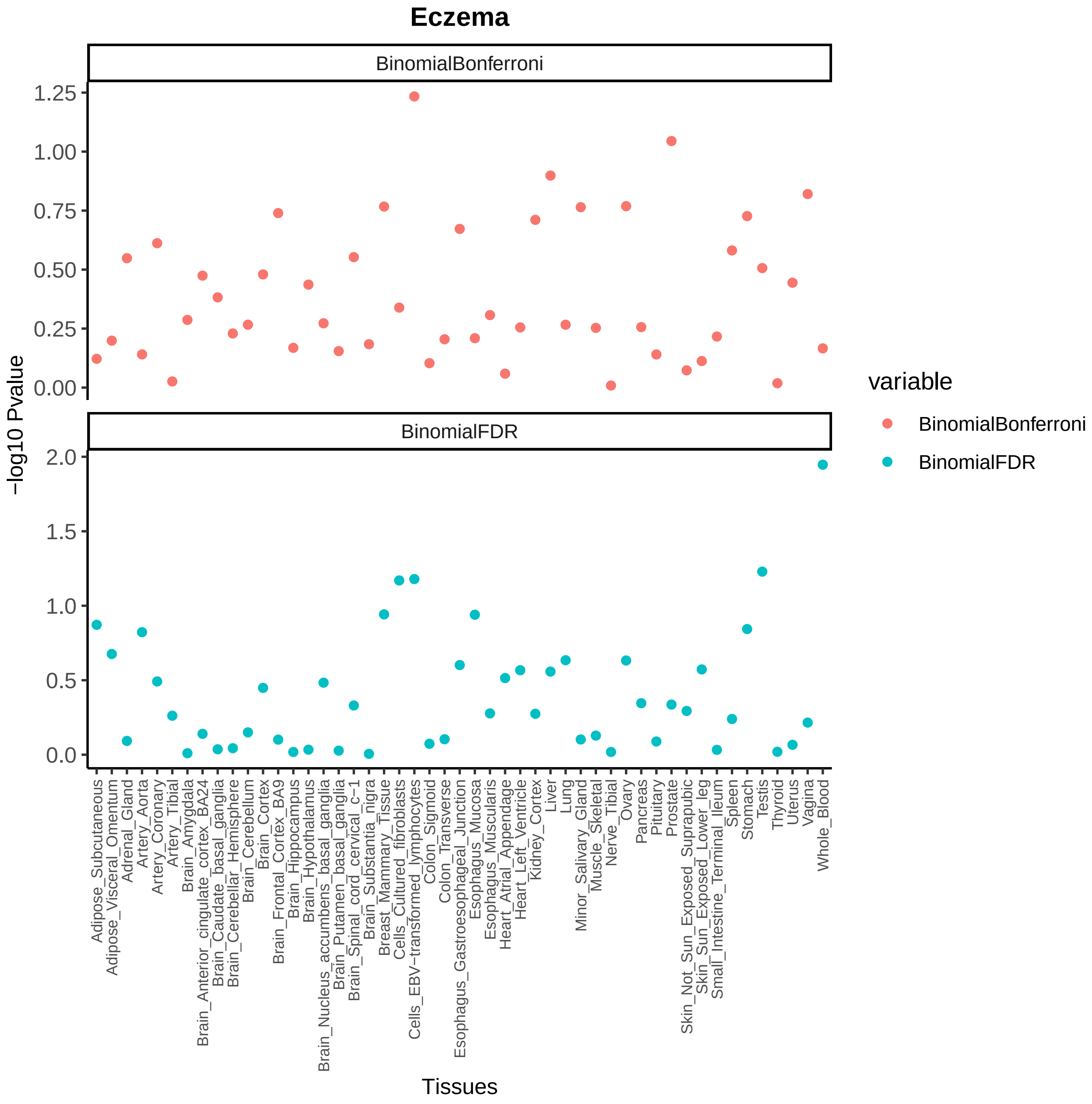

Eczema
BinomialBonferroni
BinomialFDR
-log10 Pvalue
Tissues
variable
BinomialBonferroni
BinomialFDR
0.00
0.25
0.50
0.75
1.00
1.25
0.0
0.5
1.0
1.5
2.0
Adipose_Subcutaneous
Adipose_Visceral_Omentum
Adrenal_Gland
Artery_Aorta
Artery_Coronary
Artery_Tibial
Brain_Amygdala
Brain_Anterior_cingulate_cortex_BA24
Brain_Caudate_basal_ganglia
Brain_Cerebellar_Hemisphere
Brain_Cerebellum
Brain_Cortex
Brain_Frontal_Cortex_BA9
Brain_Hippocampus
Brain_Hypothalamus
Brain_Nucleus_accumbens_basal_ganglia
Brain_Putamen_basal_ganglia
Brain_Spinal_cord_cervical_c-1
Brain_Substantia_nigra
Breast_Mammary_Tissue
Cells_Cultured_fibroblasts
Cells_EBV-transformed_lymphocytes
Colon_Sigmoid
Colon_Transverse
Esophagus_Gastroesophageal_Junction
Esophagus_Mucosa
Esophagus_Muscularis
Heart_Atrial_Appendage
Heart_Left_Ventricle
Kidney_Cortex
Liver
Lung
Minor_Salivary_Gland
Muscle_Skeletal
Nerve_Tibial
Ovary
Pancreas
Pituitary
Prostate
Skin_Not_Sun_Exposed_Suprapubic
Skin_Sun_Exposed_Lower_leg
Small_Intestine_Terminal_Ileum
Spleen
Stomach
Testis
Thyroid
Uterus
Vagina
Whole_Blood

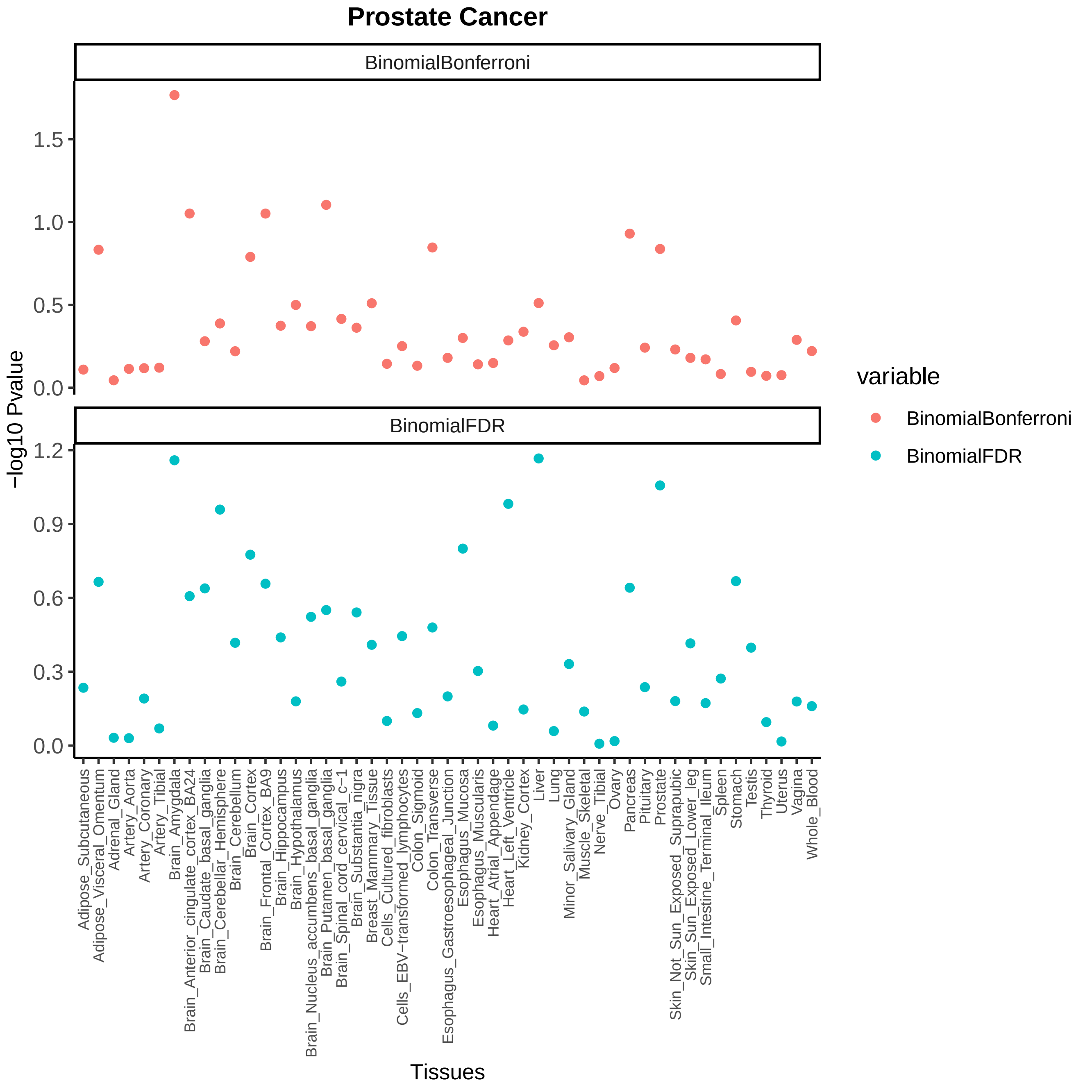

Prostate Cancer
BinomialBonferroni
BinomialFDR
−log10 Pvalue
1.5
1.0
0.5
0.0
1.2
0.9
0.6
0.3
0.0
Tissues
variable
BinomialBonferroni
BinomialFDR
Adipose_Subcutaneous
Adipose_Visceral_Omentum
Adrenal_Gland
Artery_Aorta
Artery_Coronary
Artery_Tibial
Brain_Amygdala
Brain_Anterior_cingulate_cortex_BA24
Brain_Caudate_basal_ganglia
Brain_Cerebellar_Hemisphere
Brain_Cerebellum
Brain_Cortex
Brain_Frontal_Cortex_BA9
Brain_Hippocampus
Brain_Hypothalamus
Brain_Nucleus_accumbens_basal_ganglia
Brain_Putamen_basal_ganglia
Brain_Spinal_cord_cervical_c−1
Brain_Substantia_nigra
Breast_Mammary_Tissue
Cells_Cultured_fibroblasts
Cells_EBV−transformed_lymphocytes
Colon_Sigmoid
Colon_Transverse
Esophagus_Gastroesophageal_Junction
Esophagus_Mucosa
Esophagus_Muscularis
Heart_Atrial_Appendage
Heart_Left_Ventricle
Kidney_Cortex
Liver
Lung
Minor_Salivary_Gland
Muscle_Skeletal
Nerve_Tibial
Ovary
Pancreas
Pituitary
Prostate
Skin_Not_Sun_Exposed_Suprapubic
Skin_Sun_Exposed_Lower_leg
Small_Intestine_Terminal_Ileum
Spleen
Stomach
Testis
Thyroid
Uterus
Vagina
Whole_Blood

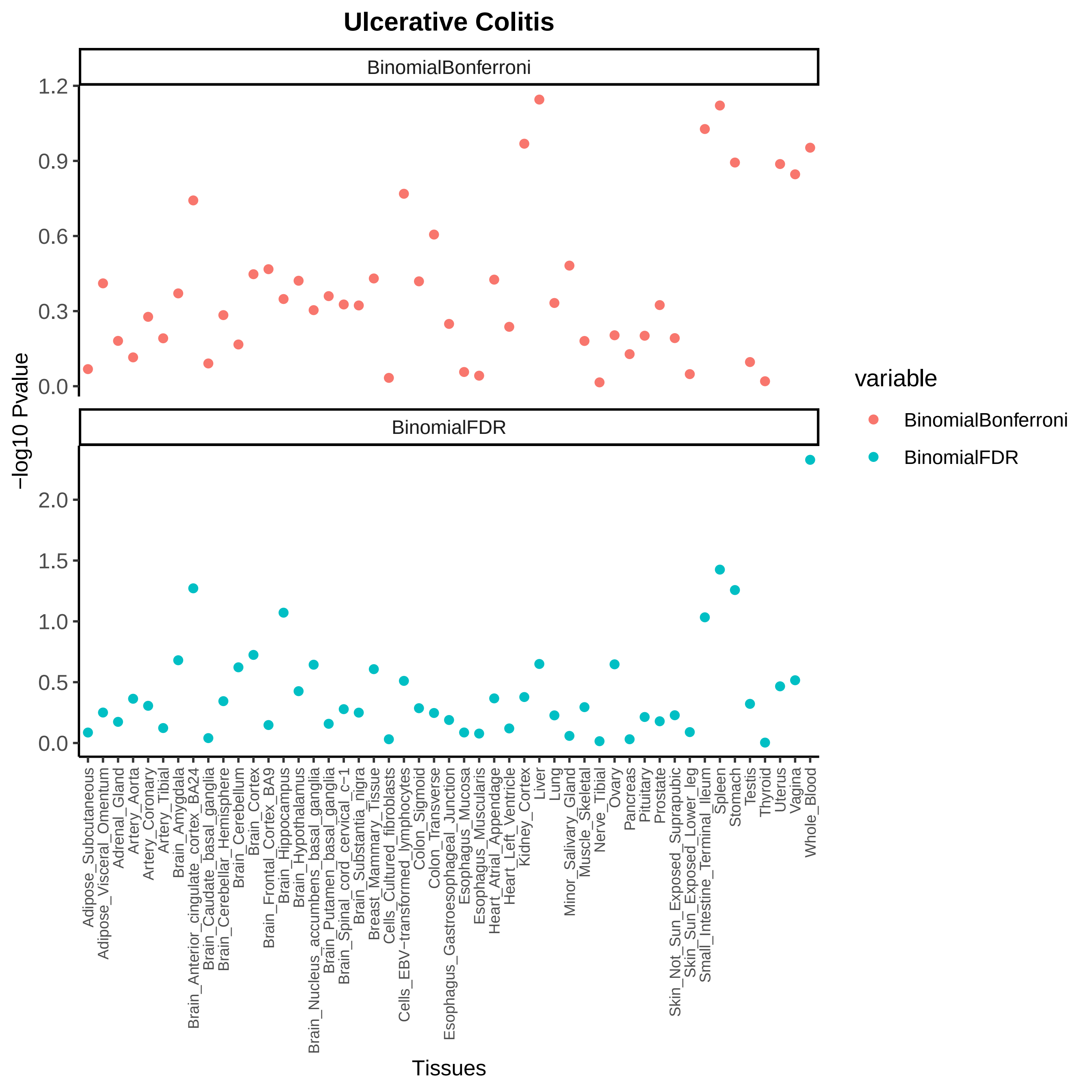

Ulcerative Colitis
BinomialBonferroni
BinomialFDR
-log10 Pvalue
Tissues
variable
BinomialBonferroni
BinomialFDR
1.2
0.9
0.6
0.3
0.0
2.0
1.5
1.0
0.5
0.0
Adipose_Subcutaneous
Adipose_Visceral_Omentum
Adrenal_Gland
Artery_Aorta
Artery_Coronary
Artery_Tibial
Brain_Amygdala
Brain_Anterior_cingulate_cortex_BA24
Brain_Caudate_basal_ganglia
Brain_Cerebellar_Hemisphere
Brain_Cerebellum
Brain_Cortex
Brain_Frontal_Cortex_BA9
Brain_Hippocampus
Brain_Hypothalamus
Brain_Nucleus_accumbens_basal_ganglia
Brain_Putamen_basal_ganglia
Brain_Spinal_cord_cervical_c-1
Brain_Substantia_nigra
Breast_Mammary_Tissue
Cells_Cultured_fibroblasts
Cells_EBV-transformed_lymphocytes
Colon_Sigmoid
Colon_Transverse
Esophagus_Gastroesophageal_Junction
Esophagus_Mucosa
Esophagus_Muscularis
Heart_Atrial_Appendage
Heart_Left_Ventricle
Kidney_Cortex
Liver
Lung
Minor_Salivary_Gland
Muscle_Skeletal
Nerve_Tibial
Ovary
Pancreas
Pituitary
Prostate
Skin_Not_Sun_Exposed_Suprapubic
Skin_Sun_Exposed_Lower_leg
Small_Intestine_Terminal_Ileum
Spleen
Stomach
Testis
Thyroid
Uterus
Vagina
Whole_Blood

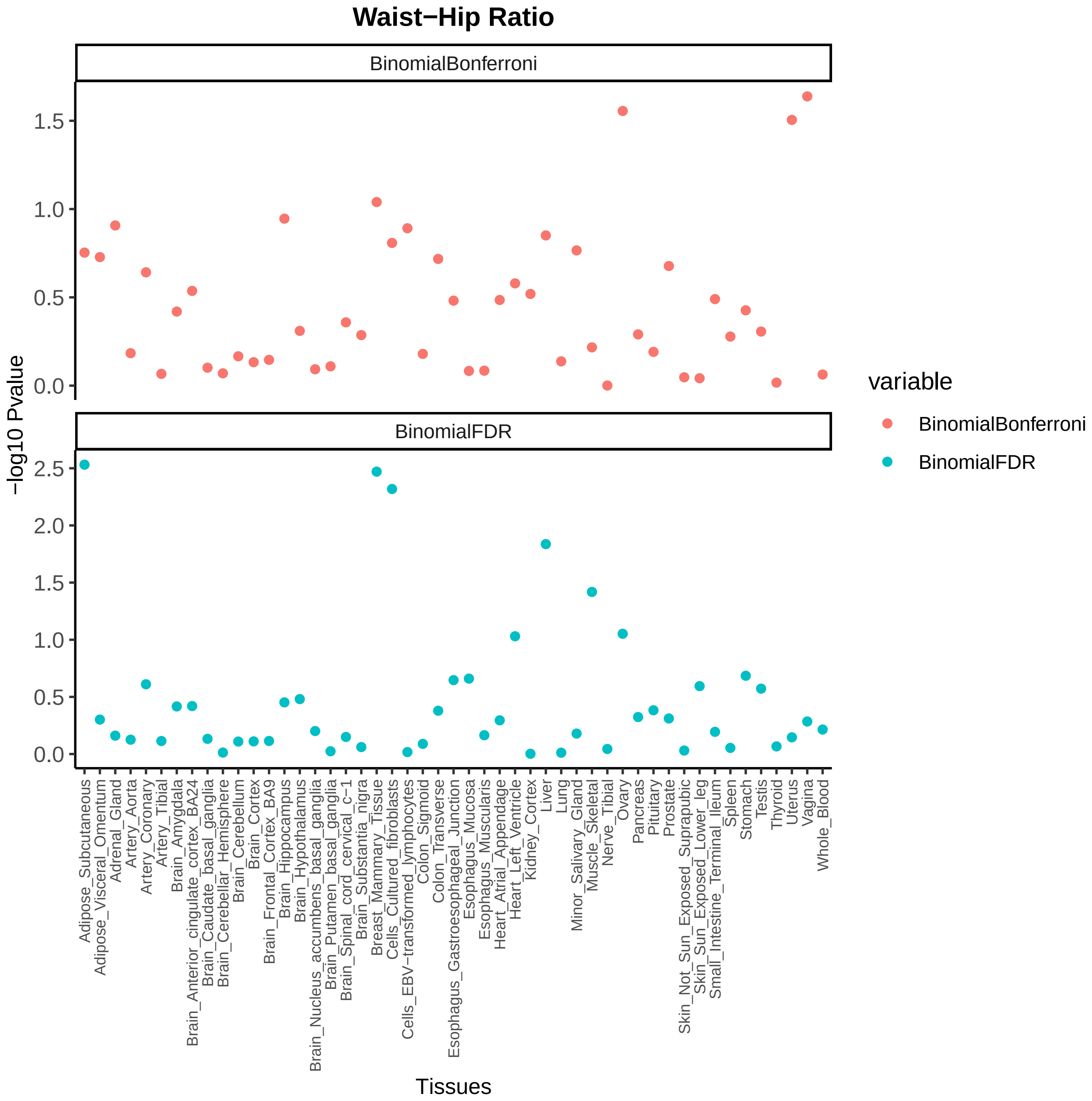
Waist−Hip Ratio
BinomialBonferroni
BinomialFDR
−log10 Pvalue
Tissues
variable
BinomialBonferroni
BinomialFDR
0.0
0.5
1.0
1.5
2.0
2.5
Adipose_Subcutaneous
Adipose_Visceral_Omentum
Adrenal_Gland
Artery_Aorta
Artery_Coronary
Artery_Tibial
Brain_Amygdala
Brain_Anterior_cingulate_cortex_BA24
Brain_Caudate_basal_ganglia
Brain_Cerebellar_Hemisphere
Brain_Cerebellum
Brain_Cortex
Brain_Frontal_Cortex_BA9
Brain_Hippocampus
Brain_Hypothalamus
Brain_Nucleus_accumbens_basal_ganglia
Brain_Putamen_basal_ganglia
Brain_Spinal_cord_cervical_c-1
Brain_Substantia_nigra
Breast_Mammary_Tissue
Cells_Cultured_fibroblasts
Cells_EBV-transformed_lymphocytes
Colon_Sigmoid
Colon_Transverse
Esophagus_Gastroesophageal_Junction
Esophagus_Mucosa
Esophagus_Muscularis
Heart_Atrial_Appendage
Heart_Left_Ventricle
Kidney_Cortex
Liver
Lung
Minor_Salivary_Gland
Muscle_Skeletal
Nerve_Tibial
Ovary
Pancreas
Pituitary
Prostate
Skin_Not_Sun_Exposed_Suprapubic
Skin_Sun_Exposed_Lower_leg
Small_Intestine_Terminal_Ileum
Spleen
Stomach
Testis
Thyroid
Uterus
Vagina
Whole_Blood

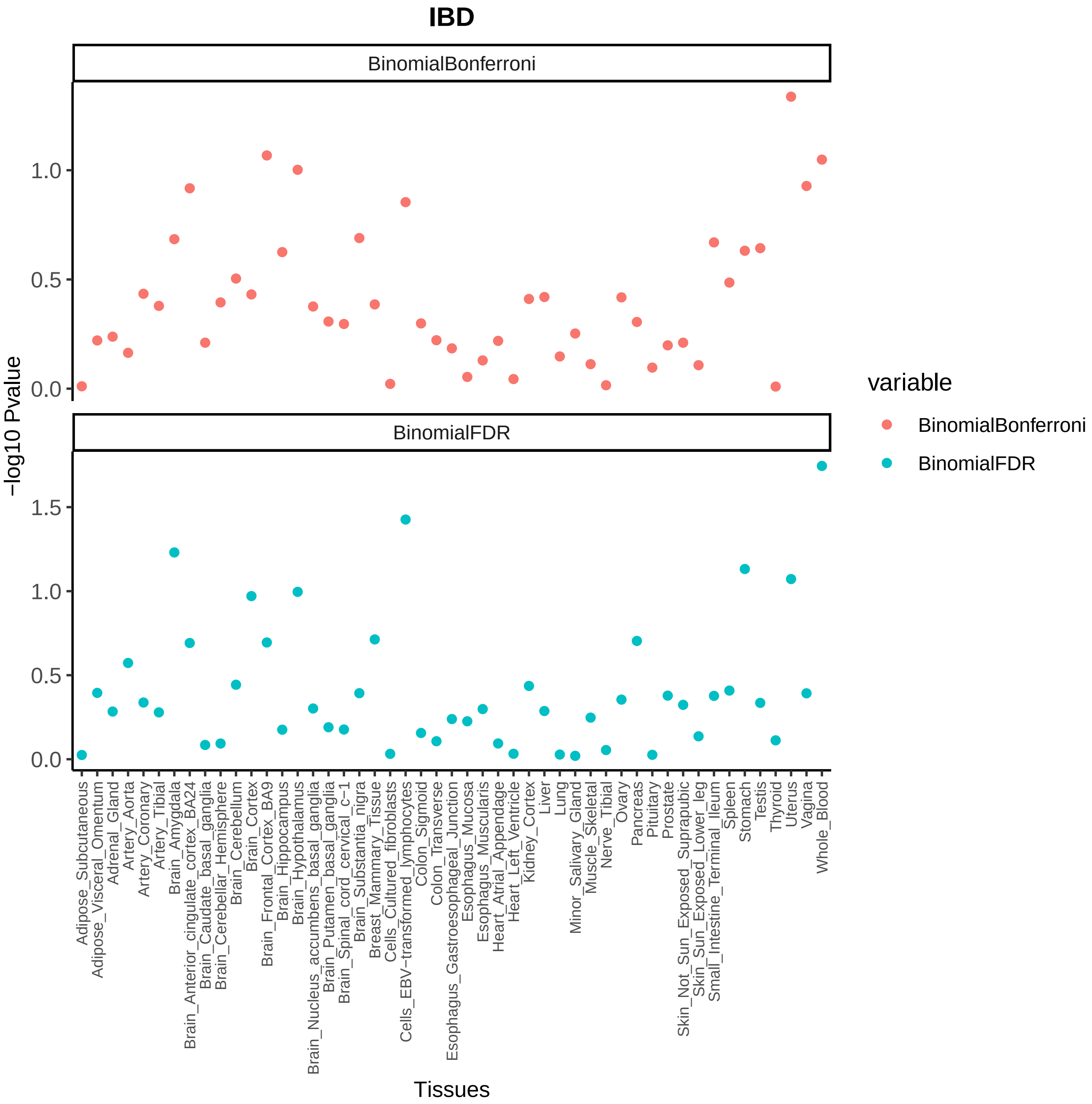

IBD
BinomialBonferroni
BinomialFDR
−log10 Pvalue
0.0
0.5
1.0
1.5
variable
BinomialBonferroni
BinomialFDR
Tissues
Adipose_Subcutaneous
Adipose_Visceral_Omentum
Adrenal_Gland
Artery_Aorta
Artery_Coronary
Artery_Tibial
Brain_Amygdala
Brain_Anterior_cingulate_cortex_BA24
Brain_Caudate_basal_ganglia
Brain_Cerebellar_Hemisphere
Brain_Cerebellum
Brain_Cortex
Brain_Frontal_Cortex_BA9
Brain_Hippocampus
Brain_Hypothalamus
Brain_Nucleus_accumbens_basal_ganglia
Brain_Putamen_basal_ganglia
Brain_Spinal_cord_cervical_c−1
Brain_Substantia_nigra
Breast_Mammary_Tissue
Cells_Cultured_fibroblasts
Cells_EBV−transformed_lymphocytes
Colon_Sigmoid
Colon_Transverse
Esophagus_Gastroesophageal_Junction
Esophagus_Mucosa
Esophagus_Muscularis
Heart_Atrial_Appendage
Heart_Left_Ventricle
Kidney_Cortex
Liver
Lung
Minor_Salivary_Gland
Muscle_Skeletal
Nerve_Tibial
Ovary
Pancreas
Pituitary
Prostate
Skin_Not_Sun_Exposed_Suprapubic
Skin_Sun_Exposed_Lower_leg
Small_Intestine_Terminal_Ileum
Spleen
Stomach
Testis
Thyroid
Uterus
Vagina
Whole_Blood

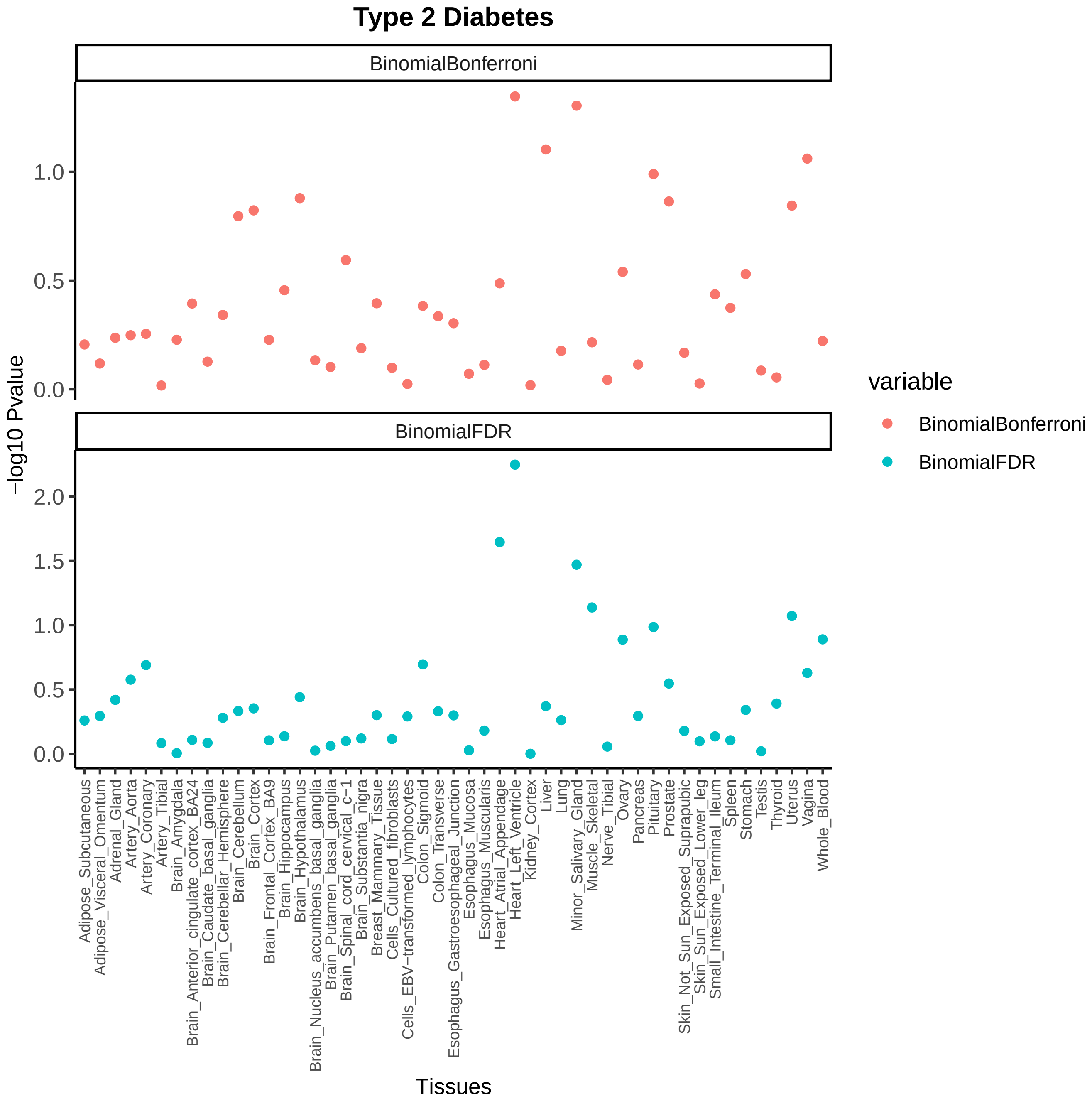

Type 2 Diabetes
BinomialBonferroni
BinomialFDR
−log10 Pvalue
0.0
0.5
1.0
1.5
2.0
Tissues
variable
BinomialBonferroni
BinomialFDR
Adipose_Subcutaneous
Adipose_Visceral_Omentum
Adrenal_Gland
Artery_Aorta
Artery_Coronary
Artery_Tibial
Brain_Amygdala
Brain_Anterior_cingulate_cortex_BA24
Brain_Caudate_basal_ganglia
Brain_Cerebellar_Hemisphere
Brain_Cerebellum
Brain_Cortex
Brain_Frontal_Cortex_BA9
Brain_Hippocampus
Brain_Hypothalamus
Brain_Nucleus_accumbens_basal_ganglia
Brain_Putamen_basal_ganglia
Brain_Spinal_cord_cervical_c−1
Brain_Substantia_nigra
Breast_Mammary_Tissue
Cells_Cultured_fibroblasts
Cells_EBV−transformed_lymphocytes
Colon_Sigmoid
Colon_Transverse
Esophagus_Gastroesophageal_Junction
Esophagus_Mucosa
Esophagus_Muscularis
Heart_Atrial_Appendage
Heart_Left_Ventricle
Kidney_Cortex
Liver
Lung
Minor_Salivary_Gland
Muscle_Skeletal
Nerve_Tibial
Ovary
Pancreas
Pituitary
Prostate
Skin_Not_Sun_Exposed_Suprapubic
Skin_Sun_Exposed_Lower_leg
Small_Intestine_Terminal_Ileum
Spleen
Stomach
Testis
Thyroid
Uterus
Vagina
Whole_Blood
