# Supplementary material for: Genome-wide imputed differential expression enrichment analysis identifies trait-relevant tissues
Source: Front Genet. 2023 Jan 6;13:1008511. doi: 10.3389/fgene.2022.1008511 (PMC9870027; doi:10.3389/fgene.2022.1008511)
Supplement: Supplementary file 8 [file DataSheet1.PDF]

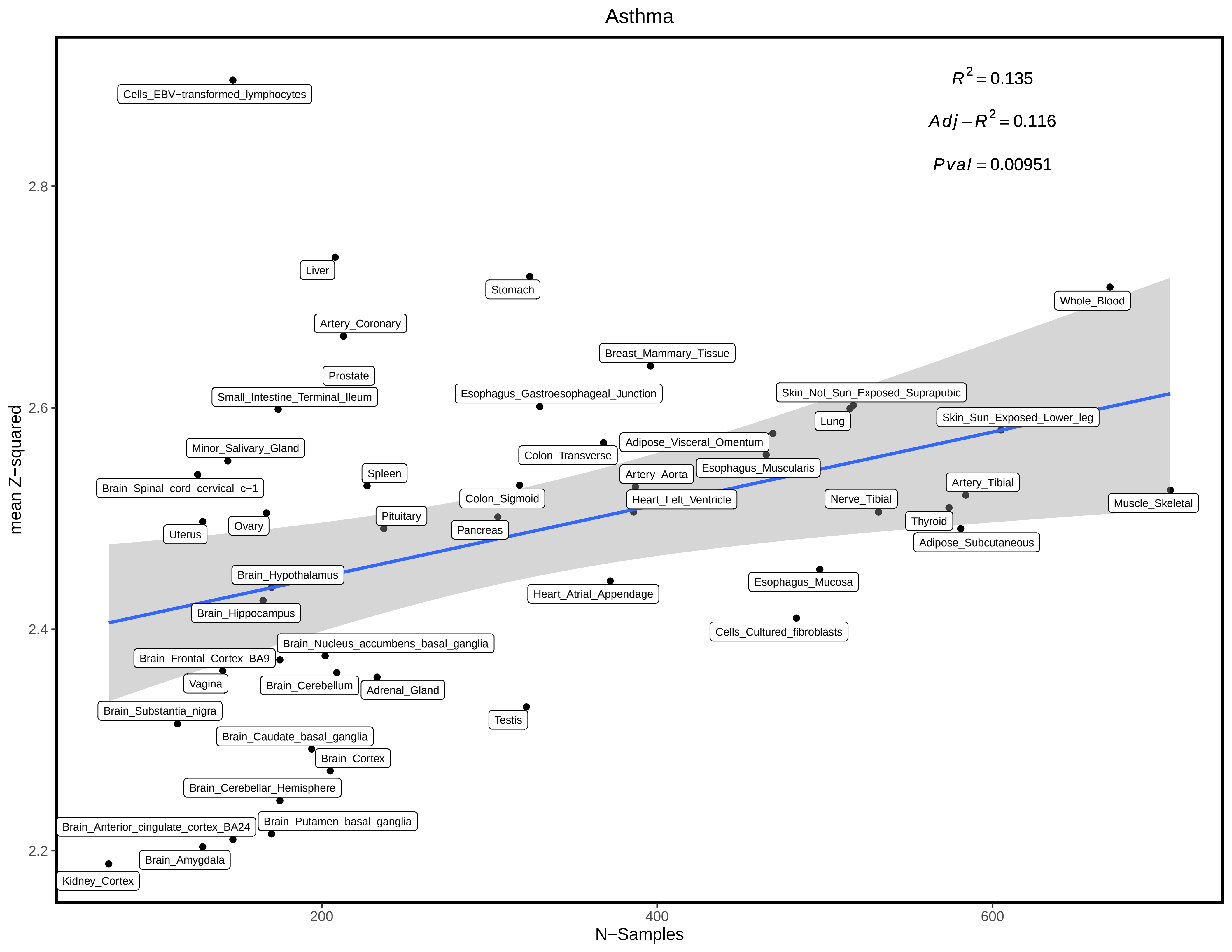

Asthma
$R^2 = 0.135$
$Adj - R^2 = 0.116$
$Pval = 0.00951$
mean Z−squared
N−Samples
2.2
2.4
2.6
2.8
200
400
600
Cells_EBV−transformed_lymphocytes
Liver
Stomach
Whole_Blood
Artery_Coronary
Breast_Mammary_Tissue
Prostate
Small_Intestine_Terminal_Ileum
Esophagus_Gastroesophageal_Junction
Skin_Not_Sun_Exposed_Suprapubic
Lung
Skin_Sun_Exposed_Lower_leg
Minor_Salivary_Gland
Colon_Transverse
Adipose_Visceral_Omentum
Spleen
Artery_Aorta
Esophagus_Muscularis
Brain_Spinal_cord_cervical_c−1
Colon_Sigmoid
Heart_Left_Ventricle
Nerve_Tibial
Artery_Tibial
Muscle_Skeletal
Pituitary
Thyroid
Uterus
Ovary
Pancreas
Adipose_Subcutaneous
Brain_Hypothalamus
Esophagus_Mucosa
Heart_Atrial_Appendage
Brain_Hippocampus
Cells_Cultured_fibroblasts
Brain_Nucleus_accumbens_basal_ganglia
Brain_Frontal_Cortex_BA9
Vagina
Brain_Cerebellum
Adrenal_Gland
Brain_Substantia_nigra
Testis
Brain_Caudate_basal_ganglia
Brain_Cortex
Brain_Cerebellar_Hemisphere
Brain_Putamen_basal_ganglia
Brain_Anterior_cingulate_cortex_BA24
Brain_Amygdala
Kidney_Cortex

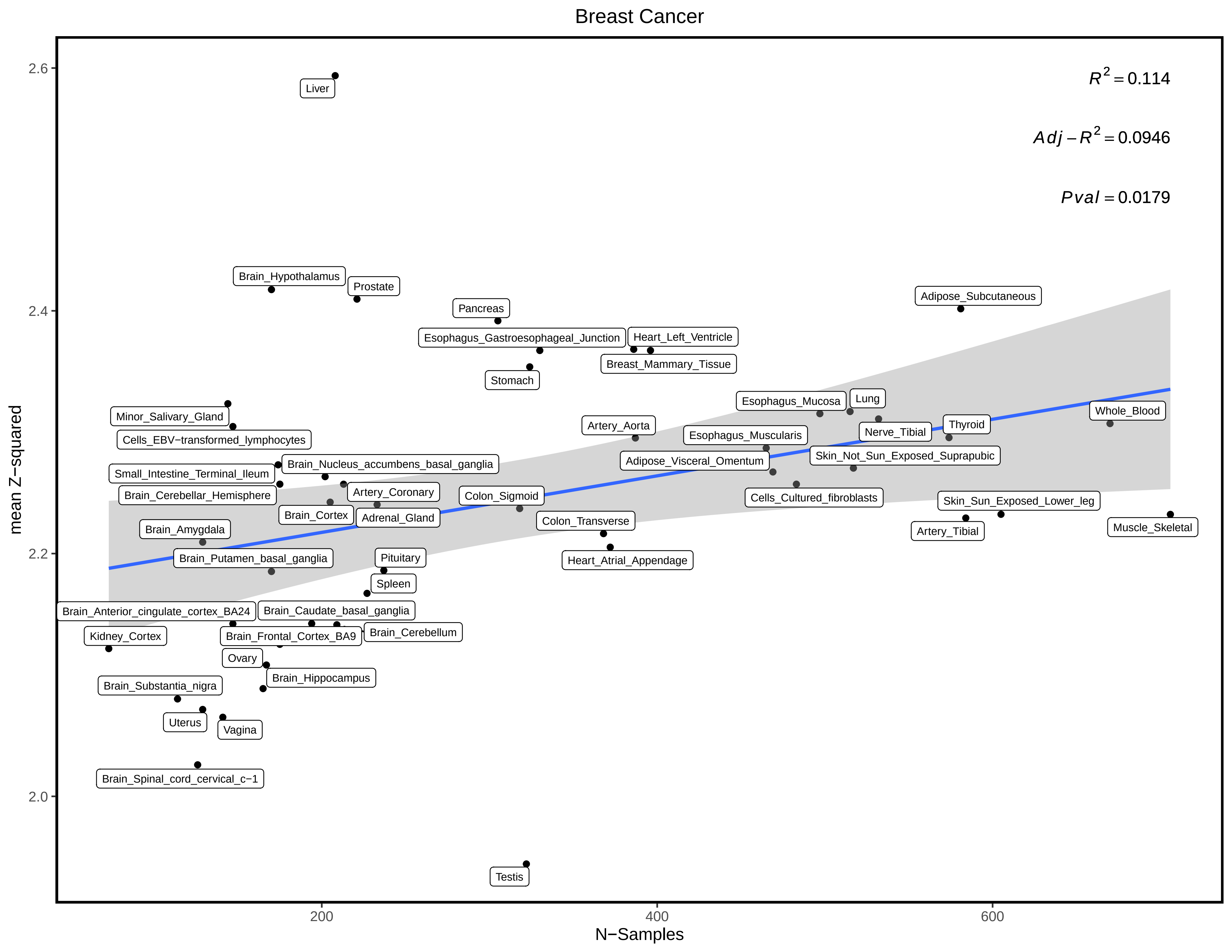

Breast Cancer
$R^2 = 0.114$
$Adj-R^2 = 0.0946$
$Pval = 0.0179$
mean Z−squared
N−Samples
2.6
2.4
2.2
2.0
200
400
600
Liver
Brain_Hypothalamus
Prostate
Pancreas
Adipose_Subcutaneous
Esophagus_Gastroesophageal_Junction
Heart_Left_Ventricle
Breast_Mammary_Tissue
Stomach
Esophagus_Mucosa
Lung
Minor_Salivary_Gland
Artery_Aorta
Whole_Blood
Nerve_Tibial
Thyroid
Cells_EBV−transformed_lymphocytes
Esophagus_Muscularis
Skin_Not_Sun_Exposed_Suprapubic
Adipose_Visceral_Omentum
Brain_Nucleus_accumbens_basal_ganglia
Small_Intestine_Terminal_Ileum
Artery_Coronary
Colon_Sigmoid
Cells_Cultured_fibroblasts
Brain_Cerebellar_Hemisphere
Skin_Sun_Exposed_Lower_leg
Brain_Cortex
Adrenal_Gland
Colon_Transverse
Muscle_Skeletal
Brain_Amygdala
Artery_Tibial
Brain_Putamen_basal_ganglia
Pituitary
Heart_Atrial_Appendage
Spleen
Brain_Anterior_cingulate_cortex_BA24
Brain_Caudate_basal_ganglia
Kidney_Cortex
Brain_Frontal_Cortex_BA9
Brain_Cerebellum
Ovary
Brain_Hippocampus
Brain_Substantia_nigra
Uterus
Vagina
Brain_Spinal_cord_cervical_c−1
Testis

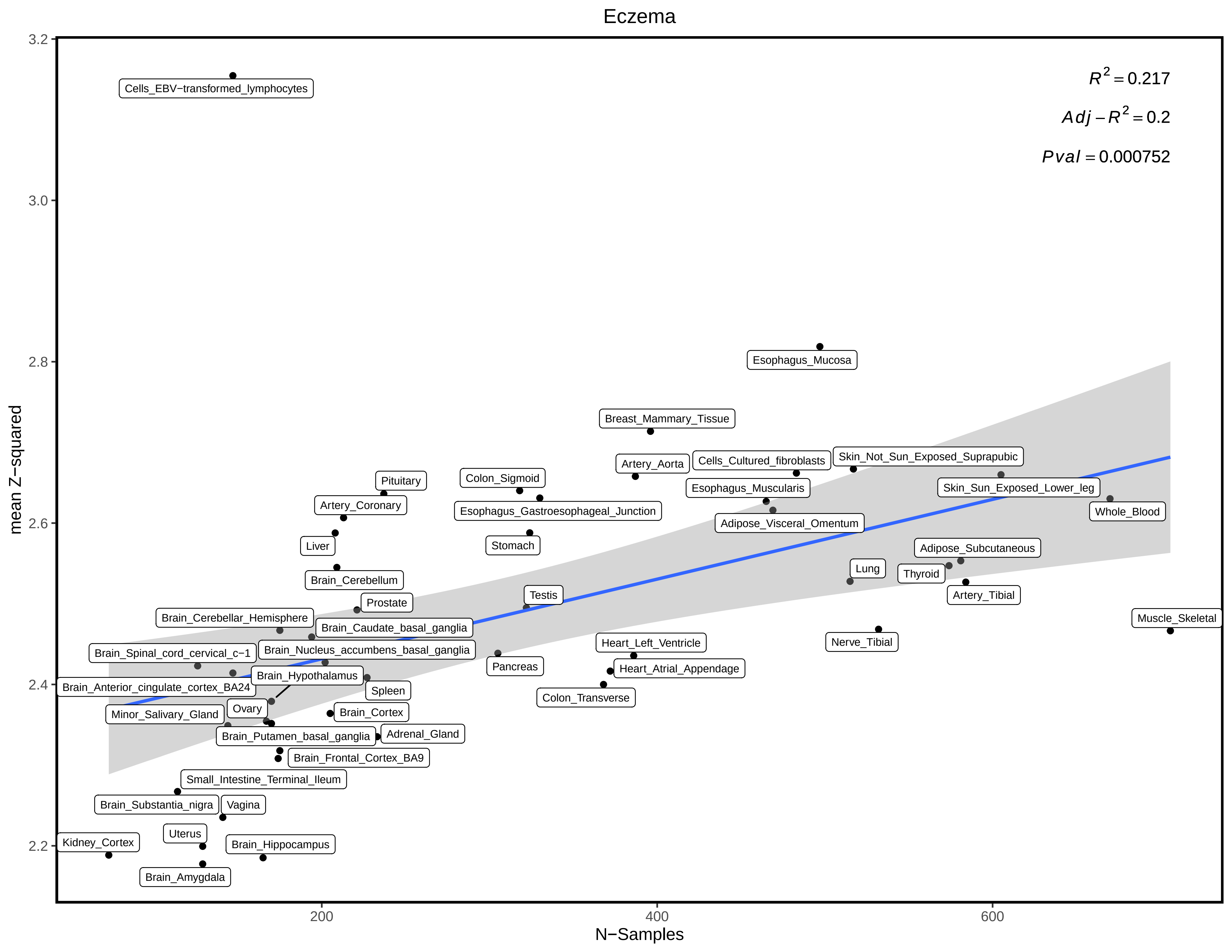

Eczema
$R^2 = 0.217$
$Adj - R^2 = 0.2$
$Pval = 0.000752$
mean Z−squared
N−Samples
3.2
3.0
2.8
2.6
2.4
2.2
200
400
600
Cells_EBV−transformed_lymphocytes
Esophagus_Mucosa
Breast_Mammary_Tissue
Artery_Aorta
Cells_Cultured_fibroblasts
Skin_Not_Sun_Exposed_Suprapubic
Skin_Sun_Exposed_Lower_leg
Whole_Blood
Esophagus_Muscularis
Adipose_Visceral_Omentum
Pituitary
Colon_Sigmoid
Artery_Coronary
Esophagus_Gastroesophageal_Junction
Liver
Stomach
Adipose_Subcutaneous
Lung
Thyroid
Artery_Tibial
Brain_Cerebellum
Testis
Prostate
Brain_Cerebellar_Hemisphere
Brain_Caudate_basal_ganglia
Muscle_Skeletal
Nerve_Tibial
Heart_Left_Ventricle
Brain_Spinal_cord_cervical_c−1
Brain_Nucleus_accumbens_basal_ganglia
Pancreas
Heart_Atrial_Appendage
Brain_Hypothalamus
Brain_Anterior_cingulate_cortex_BA24
Spleen
Colon_Transverse
Minor_Salivary_Gland
Ovary
Brain_Cortex
Brain_Putamen_basal_ganglia
Adrenal_Gland
Brain_Frontal_Cortex_BA9
Small_Intestine_Terminal_Ileum
Brain_Substantia_nigra
Vagina
Uterus
Kidney_Cortex
Brain_Hippocampus
Brain_Amygdala

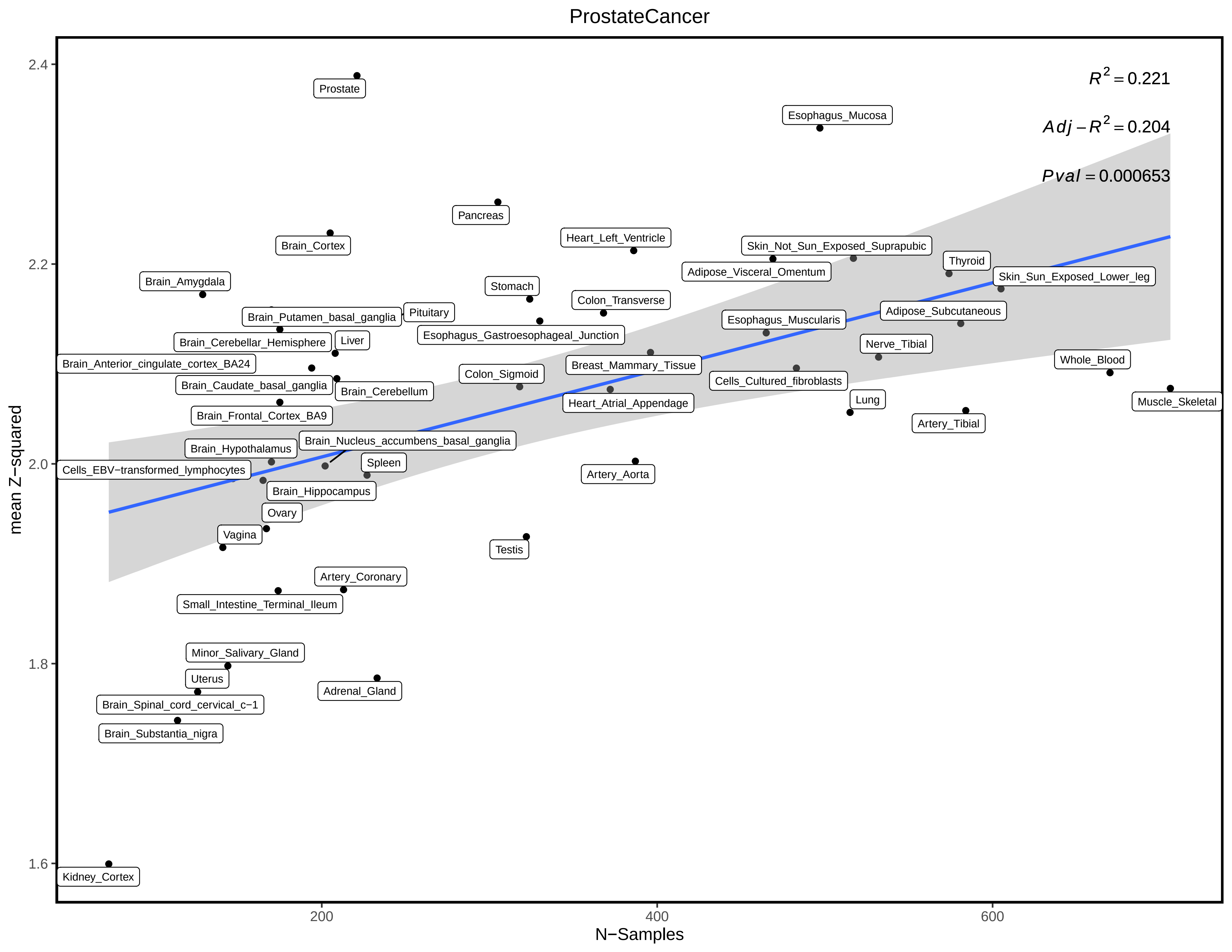
ProstateCancer
$R^2 = 0.221$
$Adj-R^2 = 0.204$
$Pval = 0.000653$
mean Z−squared
N−Samples
2.4
2.2
2.0
1.8
1.6
200
400
600
Prostate
Esophagus_Mucosa
Pancreas
Brain_Cortex
Heart_Left_Ventricle
Skin_Not_Sun_Exposed_Suprapubic
Thyroid
Adipose_Visceral_Omentum
Skin_Sun_Exposed_Lower_leg
Brain_Amygdala
Stomach
Colon_Transverse
Brain_Putamen_basal_ganglia
Pituitary
Esophagus_Muscularis
Adipose_Subcutaneous
Esophagus_Gastroesophageal_Junction
Brain_Cerebellar_Hemisphere
Liver
Nerve_Tibial
Whole_Blood
Brain_Anterior_cingulate_cortex_BA24
Breast_Mammary_Tissue
Colon_Sigmoid
Cells_Cultured_fibroblasts
Brain_Caudate_basal_ganglia
Brain_Cerebellum
Heart_Atrial_Appendage
Lung
Muscle_Skeletal
Brain_Frontal_Cortex_BA9
Artery_Tibial
Brain_Nucleus_accumbens_basal_ganglia
Brain_Hypothalamus
Spleen
Cells_EBV−transformed_lymphocytes
Artery_Aorta
Brain_Hippocampus
Ovary
Vagina
Testis
Artery_Coronary
Small_Intestine_Terminal_Ileum
Minor_Salivary_Gland
Uterus
Adrenal_Gland
Brain_Spinal_cord_cervical_c−1
Brain_Substantia_nigra
Kidney_Cortex

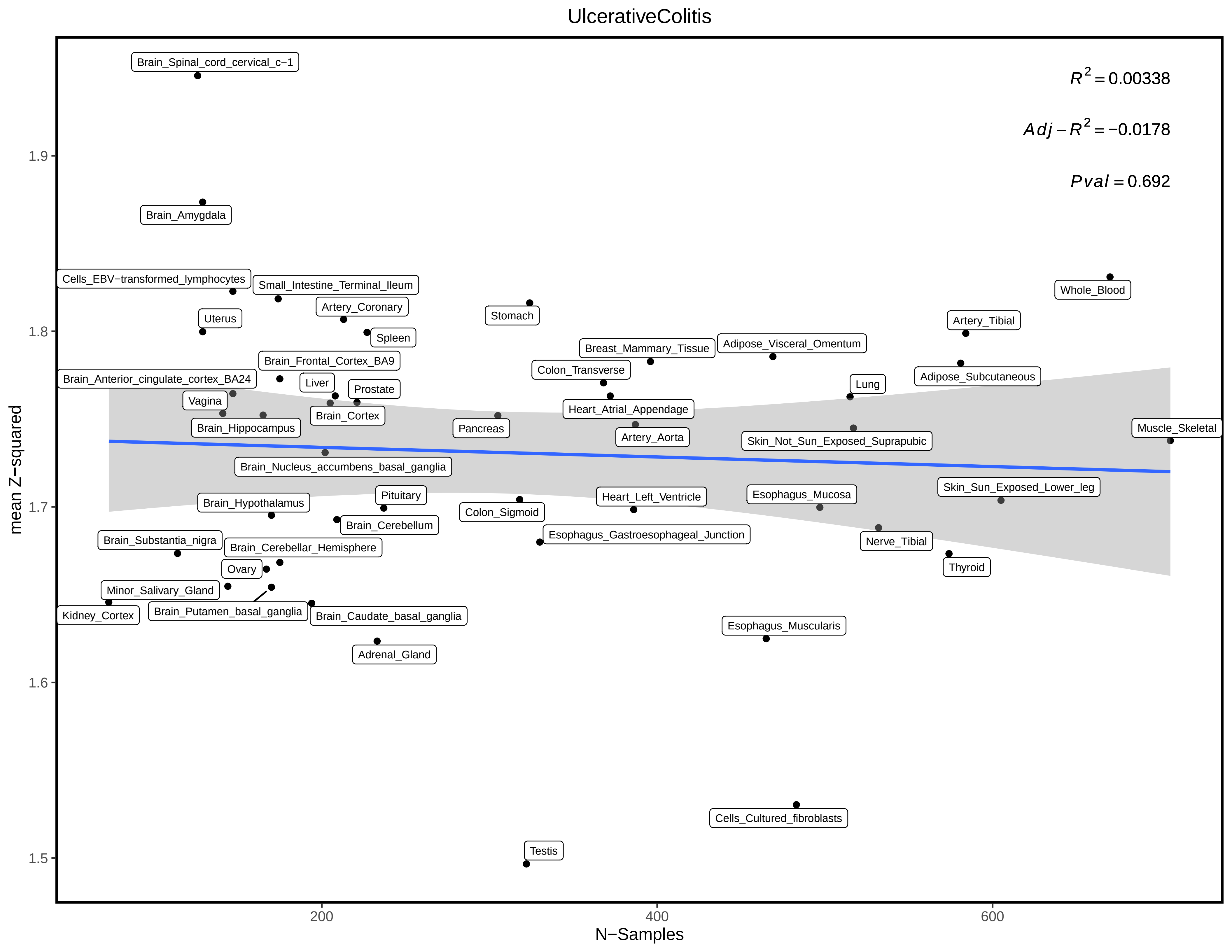

UlcerativeColitis
$R^2 = 0.00338$
$Adj-R^2 = -0.0178$
$Pval = 0.692$
mean Z−squared
N−Samples
1.5
1.6
1.7
1.8
1.9
200
400
600
Brain_Spinal_cord_cervical_c−1
Brain_Amygdala
Cells_EBV−transformed_lymphocytes
Small_Intestine_Terminal_Ileum
Artery_Coronary
Uterus
Spleen
Stomach
Whole_Blood
Artery_Tibial
Breast_Mammary_Tissue
Adipose_Visceral_Omentum
Brain_Frontal_Cortex_BA9
Colon_Transverse
Adipose_Subcutaneous
Brain_Anterior_cingulate_cortex_BA24
Liver
Prostate
Lung
Vagina
Heart_Atrial_Appendage
Brain_Cortex
Brain_Hippocampus
Pancreas
Artery_Aorta
Skin_Not_Sun_Exposed_Suprapubic
Muscle_Skeletal
Brain_Nucleus_accumbens_basal_ganglia
Pituitary
Heart_Left_Ventricle
Esophagus_Mucosa
Skin_Sun_Exposed_Lower_leg
Brain_Hypothalamus
Colon_Sigmoid
Brain_Cerebellum
Esophagus_Gastroesophageal_Junction
Nerve_Tibial
Brain_Substantia_nigra
Brain_Cerebellar_Hemisphere
Thyroid
Ovary
Minor_Salivary_Gland
Kidney_Cortex
Brain_Putamen_basal_ganglia
Brain_Caudate_basal_ganglia
Esophagus_Muscularis
Adrenal_Gland
Cells_Cultured_fibroblasts
Testis

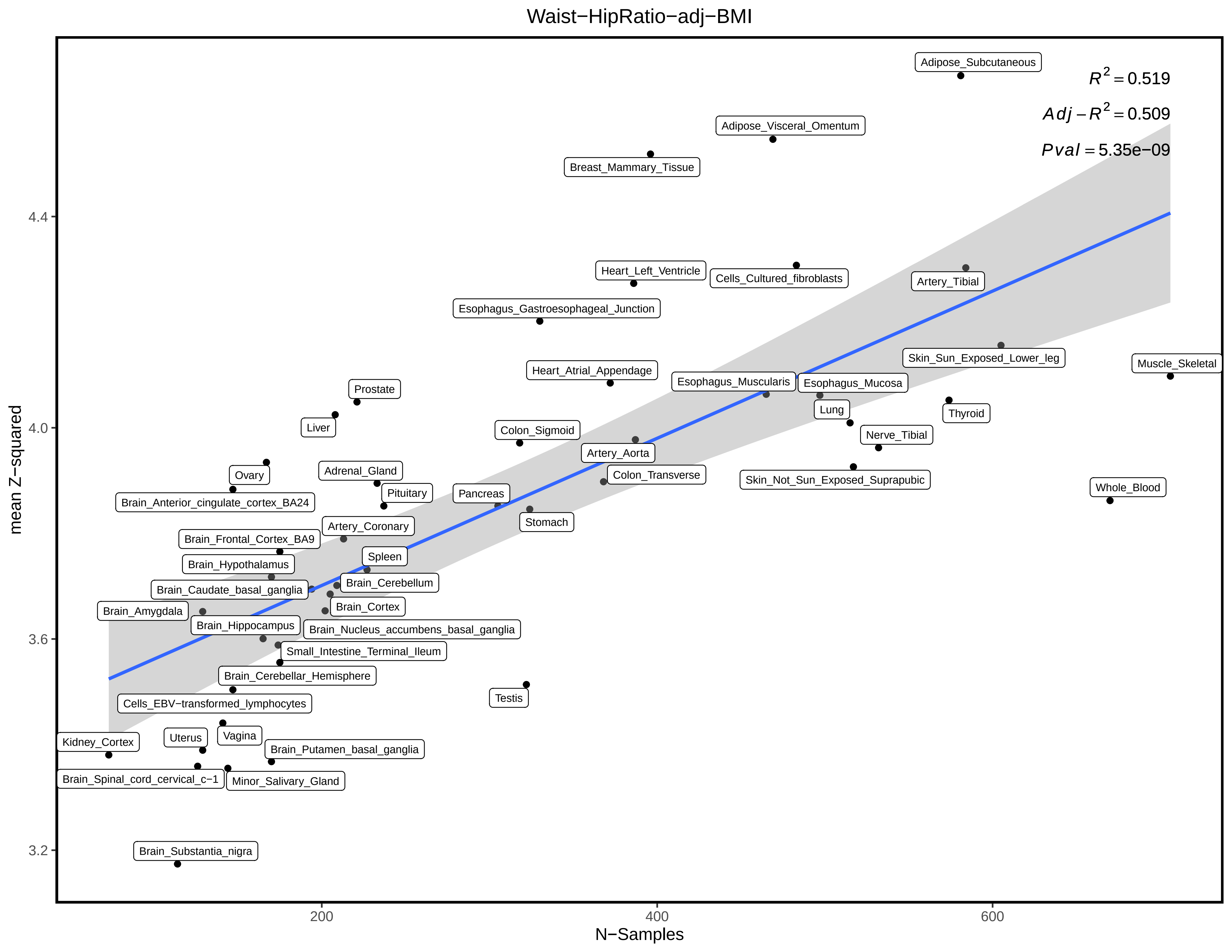

Waist−HipRatio−adj−BMI
$R^2 = 0.519$
$Adj-R^2 = 0.509$
$Pval = 5.35e-09$
mean Z−squared
N−Samples
4.4
4.0
3.6
3.2
200
400
600
Adipose_Subcutaneous
Adipose_Visceral_Omentum
Breast_Mammary_Tissue
Heart_Left_Ventricle
Cells_Cultured_fibroblasts
Artery_Tibial
Esophagus_Gastroesophageal_Junction
Skin_Sun_Exposed_Lower_leg
Muscle_Skeletal
Heart_Atrial_Appendage
Esophagus_Muscularis
Esophagus_Mucosa
Prostate
Lung
Thyroid
Liver
Colon_Sigmoid
Nerve_Tibial
Artery_Aorta
Ovary
Adrenal_Gland
Colon_Transverse
Skin_Not_Sun_Exposed_Suprapubic
Whole_Blood
Pituitary
Pancreas
Brain_Anterior_cingulate_cortex_BA24
Stomach
Artery_Coronary
Brain_Frontal_Cortex_BA9
Spleen
Brain_Hypothalamus
Brain_Cerebellum
Brain_Caudate_basal_ganglia
Brain_Cortex
Brain_Amygdala
Brain_Hippocampus
Brain_Nucleus_accumbens_basal_ganglia
Small_Intestine_Terminal_Ileum
Brain_Cerebellar_Hemisphere
Testis
Cells_EBV−transformed_lymphocytes
Uterus
Vagina
Kidney_Cortex
Brain_Putamen_basal_ganglia
Brain_Spinal_cord_cervical_c−1
Minor_Salivary_Gland
Brain_Substantia_nigra

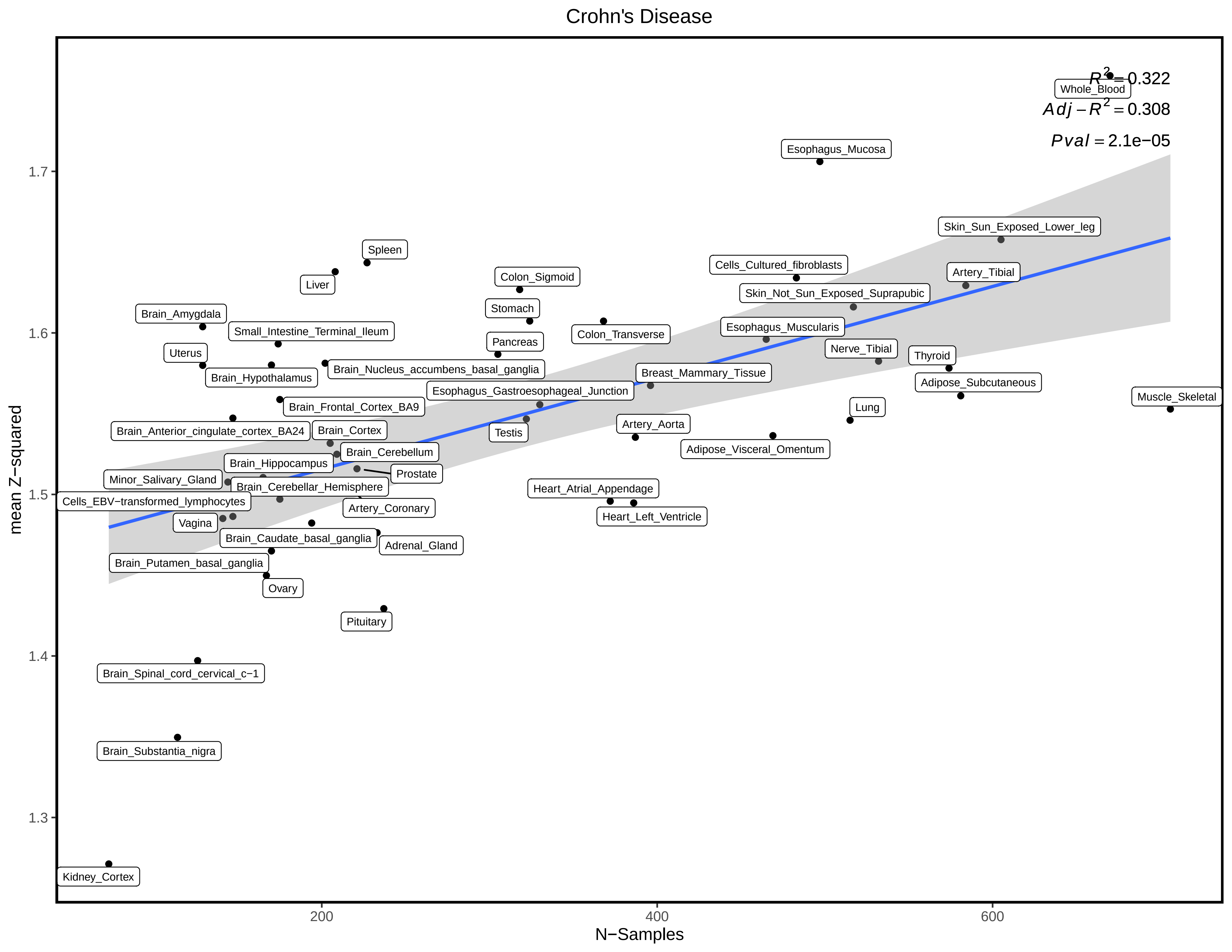

Crohn's Disease
$R^2 = 0.322$
$Adj-R^2 = 0.308$
$Pval = 2.1e-05$
mean Z−squared
N−Samples
1.3
1.4
1.5
1.6
1.7
200
400
600
Whole_Blood
Esophagus_Mucosa
Skin_Sun_Exposed_Lower_leg
Cells_Cultured_fibroblasts
Artery_Tibial
Skin_Not_Sun_Exposed_Suprapubic
Esophagus_Muscularis
Nerve_Tibial
Thyroid
Adipose_Subcutaneous
Muscle_Skeletal
Lung
Breast_Mammary_Tissue
Artery_Aorta
Adipose_Visceral_Omentum
Heart_Atrial_Appendage
Heart_Left_Ventricle
Spleen
Liver
Colon_Sigmoid
Stomach
Colon_Transverse
Pancreas
Brain_Amygdala
Small_Intestine_Terminal_Ileum
Uterus
Brain_Hypothalamus
Brain_Nucleus_accumbens_basal_ganglia
Esophagus_Gastroesophageal_Junction
Brain_Frontal_Cortex_BA9
Brain_Anterior_cingulate_cortex_BA24
Brain_Cortex
Testis
Brain_Cerebellum
Brain_Hippocampus
Prostate
Minor_Salivary_Gland
Brain_Cerebellar_Hemisphere
Cells_EBV−transformed_lymphocytes
Artery_Coronary
Vagina
Brain_Caudate_basal_ganglia
Adrenal_Gland
Brain_Putamen_basal_ganglia
Ovary
Pituitary
Brain_Spinal_cord_cervical_c−1
Brain_Substantia_nigra
Kidney_Cortex

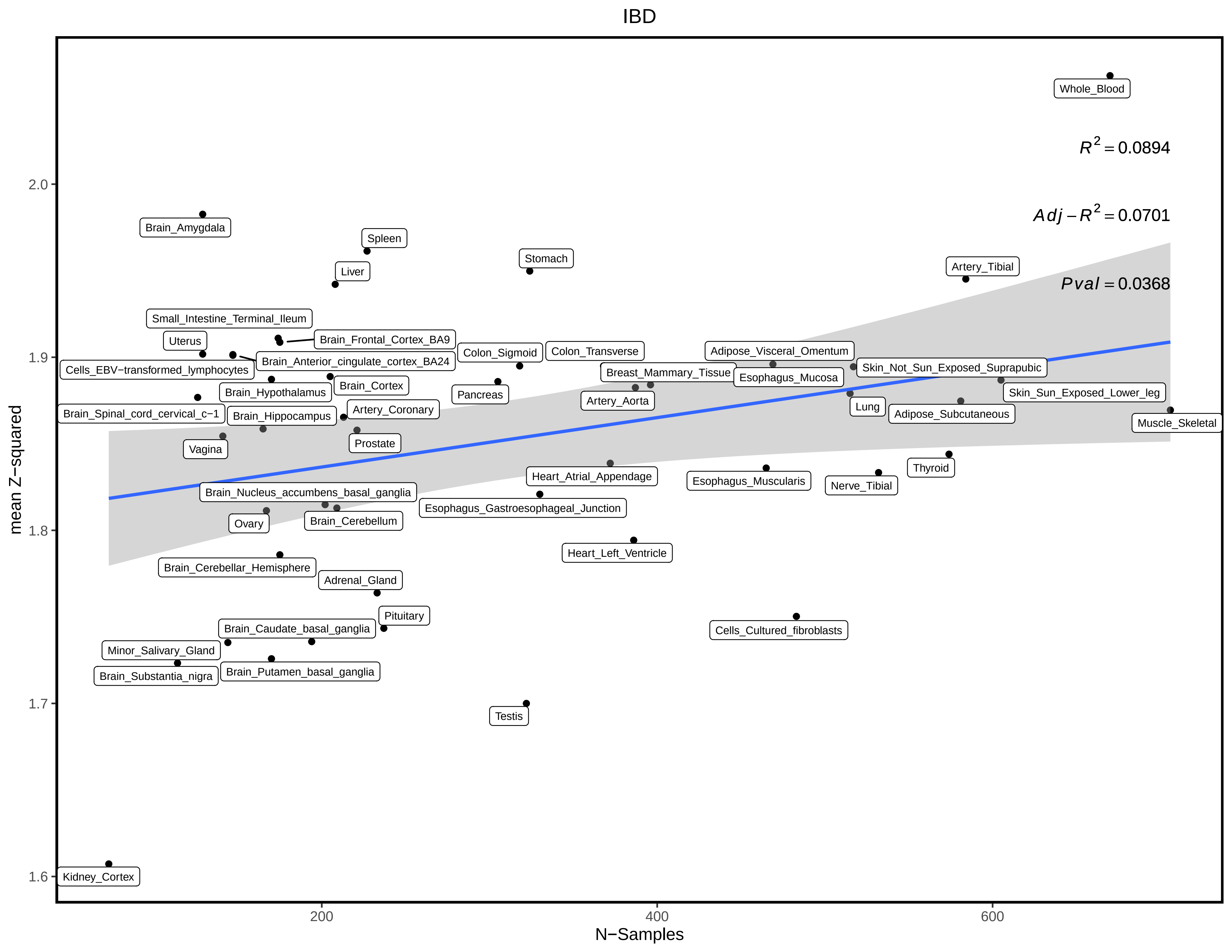

IBD
$R^2 = 0.0894$
$Adj-R^2 = 0.0701$
$Pval = 0.0368$
mean Z−squared
N−Samples
2.0
1.9
1.8
1.7
1.6
200
400
600
Whole_Blood
Brain_Amygdala
Spleen
Stomach
Artery_Tibial
Liver
Small_Intestine_Terminal_Ileum
Uterus
Brain_Frontal_Cortex_BA9
Colon_Sigmoid
Colon_Transverse
Adipose_Visceral_Omentum
Brain_Anterior_cingulate_cortex_BA24
Cells_EBV−transformed_lymphocytes
Breast_Mammary_Tissue
Skin_Not_Sun_Exposed_Suprapubic
Esophagus_Mucosa
Brain_Hypothalamus
Brain_Cortex
Pancreas
Skin_Sun_Exposed_Lower_leg
Artery_Aorta
Lung
Brain_Spinal_cord_cervical_c−1
Brain_Hippocampus
Artery_Coronary
Adipose_Subcutaneous
Muscle_Skeletal
Vagina
Prostate
Thyroid
Heart_Atrial_Appendage
Esophagus_Muscularis
Nerve_Tibial
Brain_Nucleus_accumbens_basal_ganglia
Esophagus_Gastroesophageal_Junction
Ovary
Brain_Cerebellum
Heart_Left_Ventricle
Brain_Cerebellar_Hemisphere
Adrenal_Gland
Pituitary
Brain_Caudate_basal_ganglia
Cells_Cultured_fibroblasts
Minor_Salivary_Gland
Brain_Substantia_nigra
Brain_Putamen_basal_ganglia
Testis
Kidney_Cortex

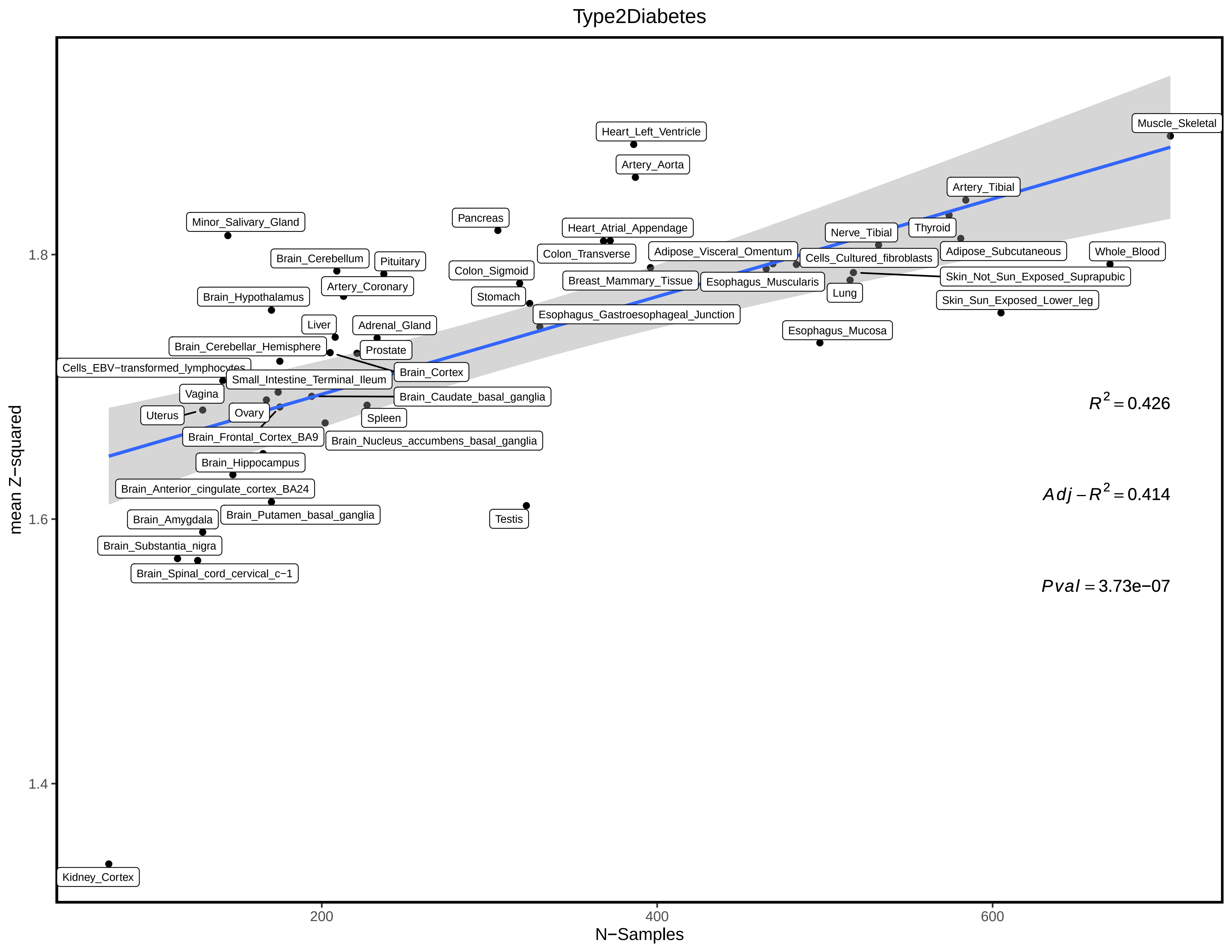

Type2Diabetes
mean Z−squared
N−Samples
1.8
1.6
1.4
200
400
600
$R^2 = 0.426$
$Adj-R^2 = 0.414$
$Pval = 3.73e-07$
Muscle_Skeletal
Heart_Left_Ventricle
Artery_Aorta
Artery_Tibial
Minor_Salivary_Gland
Pancreas
Heart_Atrial_Appendage
Nerve_Tibial
Thyroid
Brain_Cerebellum
Pituitary
Colon_Transverse
Adipose_Visceral_Omentum
Cells_Cultured_fibroblasts
Adipose_Subcutaneous
Whole_Blood
Colon_Sigmoid
Artery_Coronary
Breast_Mammary_Tissue
Esophagus_Muscularis
Skin_Not_Sun_Exposed_Suprapubic
Brain_Hypothalamus
Stomach
Lung
Skin_Sun_Exposed_Lower_leg
Esophagus_Gastroesophageal_Junction
Liver
Adrenal_Gland
Esophagus_Mucosa
Brain_Cerebellar_Hemisphere
Prostate
Cells_EBV−transformed_lymphocytes
Brain_Cortex
Small_Intestine_Terminal_Ileum
Vagina
Brain_Caudate_basal_ganglia
Uterus
Ovary
Spleen
Brain_Frontal_Cortex_BA9
Brain_Nucleus_accumbens_basal_ganglia
Brain_Hippocampus
Brain_Anterior_cingulate_cortex_BA24
Testis
Brain_Amygdala
Brain_Putamen_basal_ganglia
Brain_Substantia_nigra
Brain_Spinal_cord_cervical_c−1
Kidney_Cortex
